# Photocatalytic α-alkylation of carbamates with vinyl azaarenes

**DOI:** 10.24820/ark.5550190.p012.325

**Published:** 2025-01-25

**Authors:** Dillon R. L. Rickertsen, Emma N. George, Daniel Seidel

**Affiliations:** Center for Heterocyclic Compounds, Department of Chemistry, University of Florida, Gainesville, Florida 32611, United States

**Keywords:** Photocatalysis, C–H functionalization, acridine, azacycles, pyridine, azaarenes

## Abstract

An acridine-BF_3_ complex is a competent photocatalyst for the α-C–H bond functionalization of *N*-Boc amines. Upon the photoinduced formation of the corresponding α-carbamyl radicals, these species undergo Giese-type additions to BF_3_-activated vinyl azaarenes. Reactions tolerate a range of different azacycles and show good functional group compatibility.

## Graphical Abstract

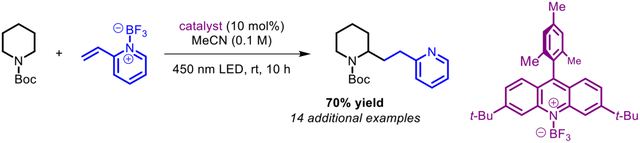

## Introduction

**Scheme 1.**
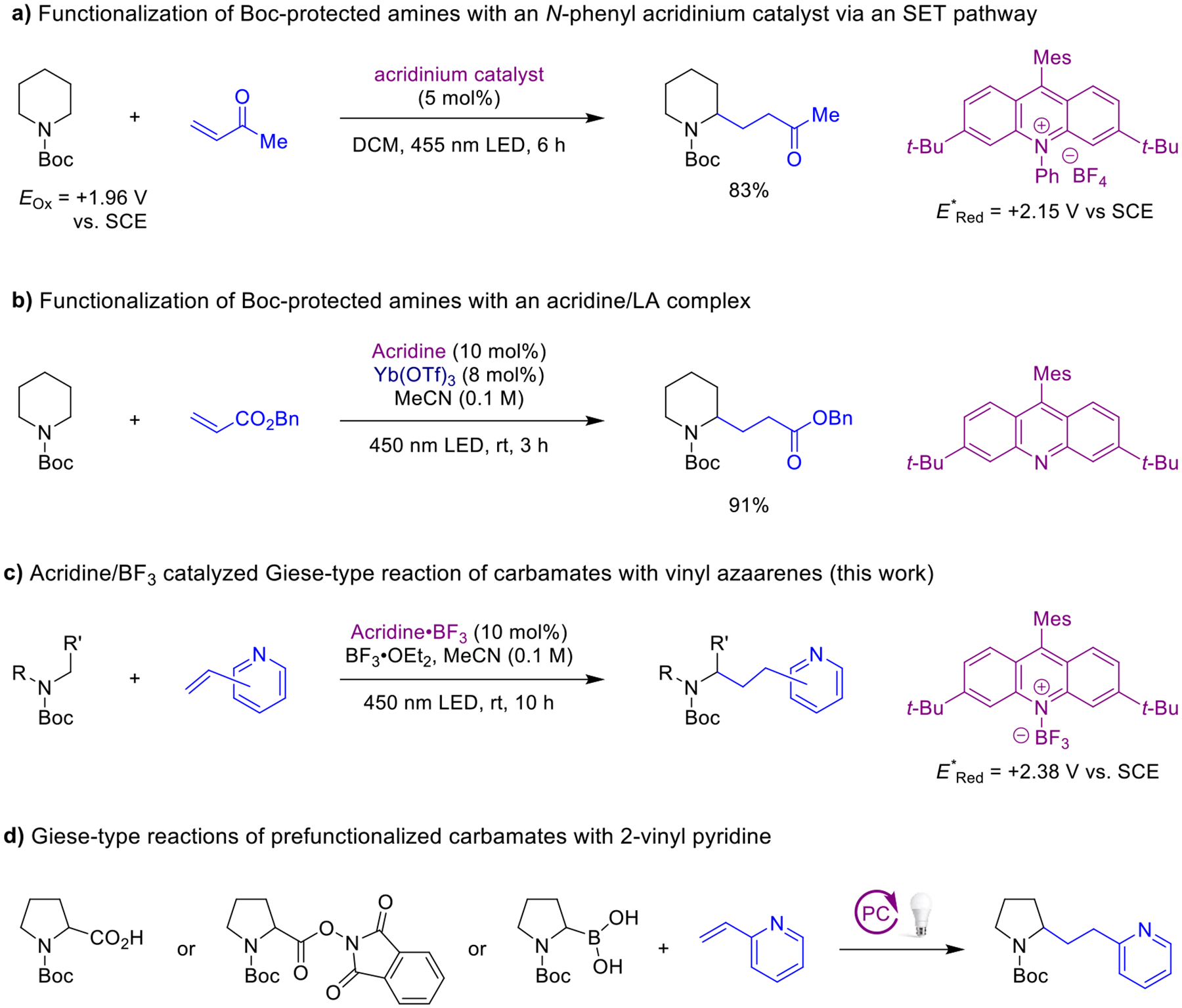
Relevant precedent and current work.

Functionalized azacycles represent highly important core structures of many bioactive substances.^1,2^ Pyridine- and piperidine-containing materials are of particular interest as they are currently the two most frequently encountered nitrogen heterocycles in FDA-approved drugs.^3^ Given the privileged status of these motifs, efforts continue to prepare more complex azacycles through diversification of simple azacycles via C–H bond functionalization, utilizing a variety of mechanistically distinct approaches.^4,5^ Photochemical transformations are especially attractive, given that they tend to operate under mild reaction conditions while typically exhibiting excellent functional group tolerance.^6,7^ While earlier photochemical approaches are often limited to readily oxidizable *N*-alkyl and *N*-aryl amines,^8–11^ more recent methods have expanded the scope to more favorable *N*-carbamoyl amines, specifically *N*-Boc (*tert*-butoxy carbonyl) protected amines, due to the ease of deprotection. Typically, photochemical approaches to the α-C–H bond functionalization of *N*-Boc amine substrates involve α-carbamyl radical intermediates that are generated via hydrogen atom transfer (HAT), a process that is facile due to the relatively low bond dissociation energies (BDEs) of the α-C–H bond.^12–20^ In contrast, α-C–H bond functionalizations of *N*-Boc amine substrates that operate via a single electron transfer (SET) oxidation/deprotonation sequence remain challenging due to the high oxidation potentials of these substrates, which are outside the reach of most common photocatalysts. In pioneering work, Nicewicz and coworkers achieved the α-C–H bond functionalization of *N*-Boc piperidine and related substrates with an *N*-phenyl acridinium catalyst possessing a large excited state reduction potential.^21,22^ α-Carbamyl radicals generated via a SET pathway were shown to undergo Giese reactions^23^ with a range of acceptors (Scheme 1a). While acridinium-type photocatalyst are wildly popular,^24–26^ acridine photocatalysts are gaining popularity.^27–40^ We recently reported a strategy that expands the scope of these Giese reactions to more challenging amine substrates and less electrophilic conjugate acceptors such as simple acrylates (Scheme 1b).^41^ This method involves the use of a photoactive complex formed in situ from an acridine and a Lewis acid,^42,43^ a modular approach that allows for dialing in the excited state reduction potential (+2.07–2.38 V vs. SCE) of the catalyst. Here we report an extension of this concept by employing vinyl azaarenes as conjugate acceptors (Scheme 1c). These reactions are attractive in that they generate products that contain two common azacyclic pharmacophores.

Vinyl azaarenes such as vinyl pyridines have been utilized in a range of photochemical Giese-type reactions, engaging with a variety of different alkyl radicals.^44–52^ Previous photocatalytic methods for the addition of α-carbamyl radicals to vinyl azaarenes are limited to prefunctionalized carbamate substrates (Scheme 1d).^53–57^ For instance, Sparling and co-workers developed a photoinduced decarboxylation of *N*-Boc proline to generate the corresponding α-carbamyl radical which was then trapped with 2-vinylpyridine.^53^ The König group employed a decarboxylation strategy involving redox active esters.^54^ A similar approach was later utilized by Lin and coworkers.^55^ Sharma and co-workers exploited α-boronic acids as carbamyl radical precursors.^56^ Overall, the existing photochemical methods exhibit limited substrate scope with respect to the carbamyl radical precursors.

## Results and Discussion

**Scheme 2.**
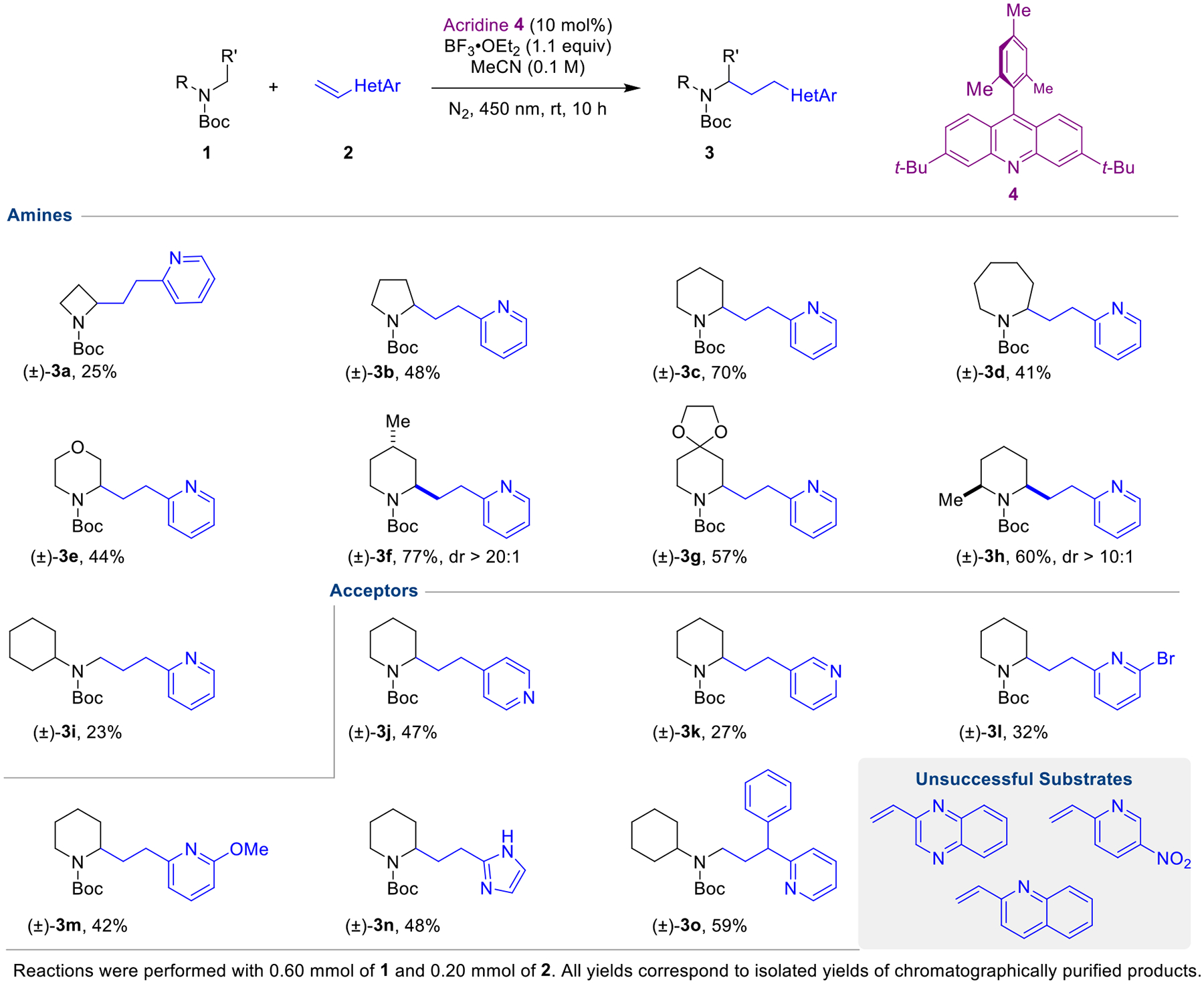
Scope of the reaction.

**Scheme 3.**
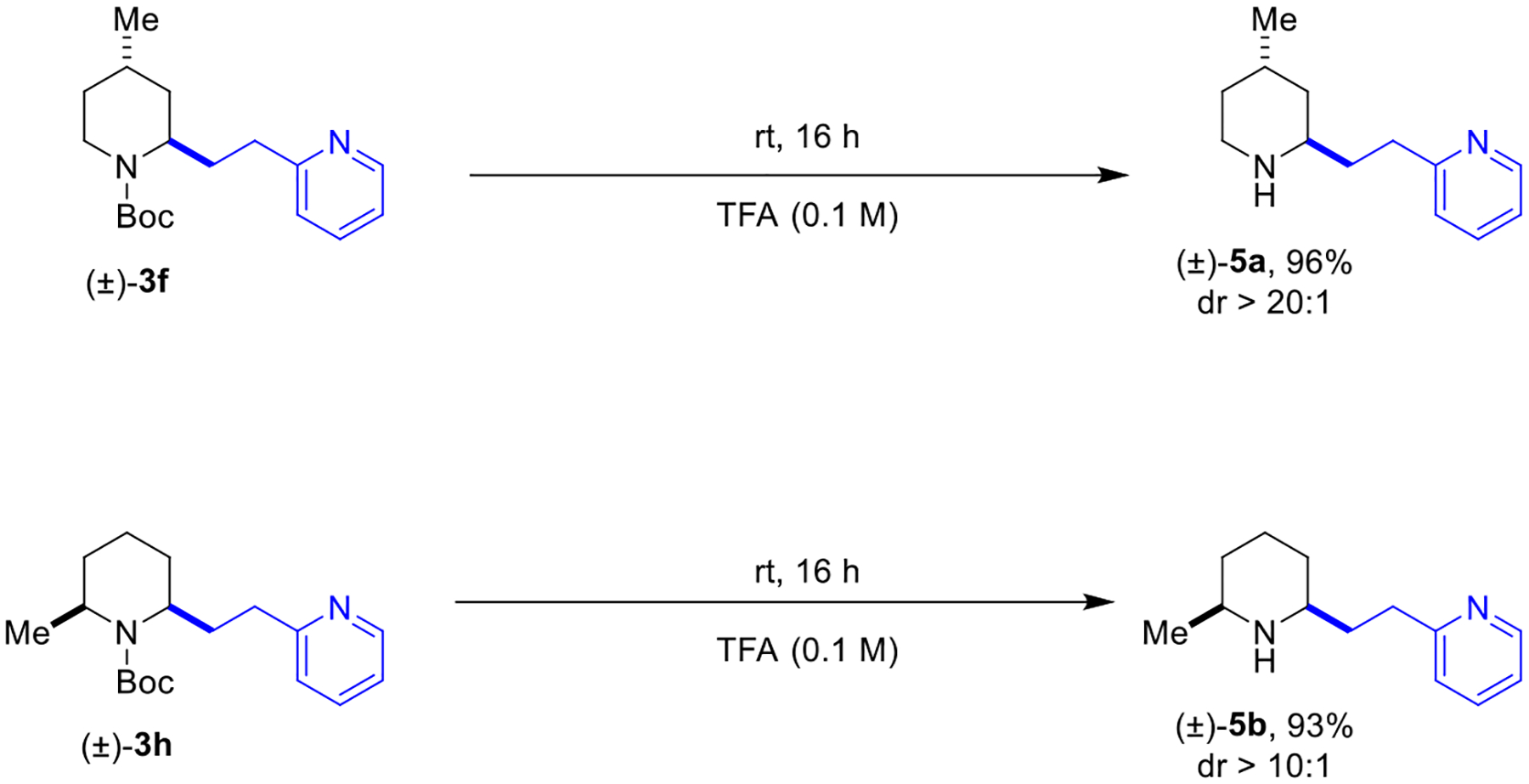
Deprotection of selected products.

Our previous study on the acridine/Lewis acid catalyzed α-C–H bond functionalization of carbamates focused on Giese reactions of photochemically generated carbamyl radicals with α,β-unsaturated esters.^41^ In an extension of this concept, we also reported a single example of a Giese-type reaction of *tert*-butyl 4-benzylpiperidine-1-carboxylate with 2-vinylpyridine. In this case, it proved essential to employ an excess of boron trifluoride etherate as an additive, the role of which is twofold: 1) generate the active photocatalyst, and 2) activate 2-vinylpyridine toward addition. To explore the scope and limitations of this transformation, we evaluated a range of carbamate donors **1** and vinyl azaarene acceptors **2** (Scheme 2). A variety of *N*-Boc amines engaged 2-vinylpyridine upon irradiation with 450 nm LED light in presence of acridine **4** and excess boron trifluoride etherate. While the yields are variable, a range of different ring sizes were tolerated (products **3a**–**d**). *N*-Boc morpholine also underwent the title reaction to provide product **3e**. Piperidine rings containing substituents at the C4- and C6-positions furnished products **3f** and **3h** in good yields and excellent diastereoselectivities. These reactions presumably proceed via a Fürst-Plattner-type transition state, highly favoring one diastereomer as seen in related reactions (not shown).^21,41^ An acid-labile ketal functionality (product **3g**) and linear amines (product **3i**) were also tolerated. Next, we explored azaarene acceptors. 4-Vinylpyridine performed well (product **3j**). Interestingly, 3-vinylpyridine also furnished the corresponding product **3k**, albeit in low yield. *N*-Boc piperidine engaged differently substituted vinyl pyridines to provide halogen-substituted product **3l** and methoxy-substituted product **3m**. Regarding different types of acceptors, 2-vinylimidazole was identified as a viable substrate (product **3n**). As an example of a 1,1-disubstituted acceptor, 2-(1-phenylvinyl)pyridine readily underwent the title reaction (product **3o**). Current substrate limitations include fused heterocycles such as vinyl quinoxaline and vinyl quinoline. While the reasons for the failure of these substrates to undergo the title reaction remain unclear, it appears that, at least in some cases, polymerization of the acceptors represents one of the potential decomposition pathways. Product deprotection was readily accomplished as illustrated in two representative examples (Scheme 3).

## Conclusions

In summary, a photoactive acridine-BF_3_ complex was shown to catalyze the addition of various *N*-Boc amines to vinyl azaarenes, achieving α-C–H bond functionalization under mild conditions. This approach allows for the facile synthesis of small molecules containing multiple nitrogen heterocycles. These materials are likely of interest to medicinal chemistry programs.

## Experimental Section

### General:

Starting materials and reagents were purchased from commercial sources and used as received unless stated otherwise. Anhydrous acetonitrile (MeCN) was dried using a mBraun solvent system. Purification of reaction products was carried out by flash column chromatography using Sorbent Technologies Standard Grade silica gel (60 Å, 230–400 mesh). Analytical thin layer chromatography was performed on EM Reagent 0.25 mm silica gel 60 F_254_ plates. Visualization was accomplished with UV light, potassium permanganate, and Dragendorff-Munier stains followed by heating. Proton nuclear magnetic resonance spectra (^1^H NMR) were recorded on a Bruker Avance HD II spectrometer operating at 400 MHz or a Bruker Avance HD II operating at 600 MHz instrument and chemical shifts are reported in ppm using the solvent as an internal standard (CDCl_3_ at 7.26 ppm). Data are reported as app = apparent, s = singlet, d = doublet, t = triplet, q = quartet, qt = quintet, dd = doublet of doublets, td = triplet of doublets, m = multiplet; coupling constant(s) in Hz. Proton-decoupled carbon nuclear magnetic resonance spectra (^13^C NMR) spectra were recorded on a Bruker Avance HD II spectrometer operating at 400 MHz or Bruker Avance HD II operating at 600 MHz instrument and chemical shifts are reported in ppm using the solvent as an internal standard (CDCl_3_ at 77.16 ppm). Some NMR signals are broad (br) due to the time scale of the rotation about the N–CO bond. Most compounds are a mixture of rotamers. High resolution mass spectra (HRMS) were obtained from the Mass Spectrometry Core Laboratory of the University of Florida (Agilent 6230 ESI-TOF instrument). Photochemical reactions were carried out using a PennPhD Photoreactor M2 with a 450 nm LED. The light source was operated at an intensity level of 85% and a stir rate of 350 rpm was applied. Boc-protected substrates were purchased from commercial sources or prepared according to literature procedures.^41^ The following substrates were prepared according to literature procedures and characterization data matched our own in all regards: 3-vinylpyridine,^58^ 2-bromo-6-vinylpyridine,^59^ 2-methoxy-6-vinylpyridine,^60^ 2-(1-phenylvinyl)pyridine,^60^ and 2-vinyl-1*H*-imidazole.^61^ 3,6-di-*tert*-butyl-9-mesitylacridine (**4**) was prepared according to our previous reports.^38,41^

### General Procedure A (Reactions with 2-Vinylpyridine):

To a flame dried vial was added a stir bar, 3,6-di-*tert*-butyl-9-mesitylacridine (8.2 mg, 0.02 mmol, 0.1 equiv), and Boc-amine (0.6 mmol, 3 equiv). Anhydrous MeCN (2 mL) was added, and the vial was sealed with a rubber septum. Nitrogen gas was bubbled through the reaction mixture for 5 minutes. After purging with nitrogen, 2-vinyl pyridine (21.5 μL, 0.2 mmol, 1 equiv) was added followed by BF_3_·OEt_2_ (27.0 μL,0.22 mmol, 1.1 equiv) resulting in a bright yellow solution. The septum was wrapped in parafilm. The reaction vial was irradiated with 450 nm light (85% intensity) at room temperature for 10 hours. Following irradiation, 1 M NaOH (2 mL) was added, and the reaction was vigorously stirred for 30 minutes and then extracted with DCM (3 × 3 mL). The combined organic layers were dried over Na_2_SO_4_. The dried organic layer was filtered, and the solvent was removed under reduced pressure. The resulting residue was purified using silica gel chromatography.

### General Procedure B (Liquid Vinyl Azaarenes):

To a flame dried vial was added a stir bar, 3,6-di-*tert*-butyl-9-mesitylacridine (8.2 mg, 0.02 mmol, 0.1 equiv), and Boc-amine (0.6 mmol, 3equiv). Anhydrous MeCN (2 mL) was added, and the vial was sealed with a rubber septum. Nitrogen gas was bubbled through the reaction mixture for 5 minutes. After purging with nitrogen, radical acceptor (0.2 mmol, 1 equiv) was added followed by BF_3_·OEt_2_ (27.0 μL,0.22 mmol, 1.1 equiv) resulting in a bright yellow solution. The septum was wrapped in parafilm. The reaction vial was irradiated with 450 nm light (85% intensity) at room temperature for 10 hours. Following irradiation 1 M NaOH (2 mL) was added, and the reaction was vigorously stirred for 30 minutes and then extracted with DCM (3 × 3 mL). The combined organic layers were dried over Na_2_SO_4_. The dried organic layer was filtered, and the solvent was removed under reduced pressure. The resulting residue was purified using silica gel chromatography.

### General Procedure C (Solid Vinyl Azaarenes):

To a flame dried vial was added a stir bar, 3,6-di-*tert*-butyl-9-mesitylacridine (8.2 mg, 0.02 mmol, 0.1 equiv), Boc-amine (0.6 mmol, 3equiv), and radical acceptor (0.2 mmol, 1 equiv). Anhydrous MeCN (2 mL) was added, and the vial was sealed with a rubber septum. Nitrogen gas was bubbled through the reaction mixture for 5 minutes. After purging with nitrogen, BF_3_·OEt_2_ (27.0 μL, 0.22 mmol, 1.1 equiv) was added resulting in a bright yellow solution. The septum was wrapped in parafilm. The reaction vial was irradiated with 450 nm light (85% intensity) at room temperature for 10 hours. Following irradiation 1 M NaOH (2 mL) was added, and the reaction was vigorously stirred for 30 minutes and then extracted with DCM (3 × 3 mL). The combined organic layers were dried over Na_2_SO_4_. The dried organic layer was filtered, and the solvent was removed under reduced pressure. The resulting residue was purified using silica gel chromatography.

### *tert*-Butyl 2-[2-(pyridin-2-yl)ethyl]azetidine-1-carboxylate (3a):

Following general procedure A, compound (±)-**3a** was obtained from *tert*-butyl azetidine-1-carboxylate (94.3 mg, 0.6 mmol, 3 equiv) and 2-vinylpyridine (21.5 μL, 0.2 mmol, 1 equiv) as a light yellow oil in 25% yield (13.1 mg). Hexane containing ethyl acetate (25–80%) was used as the eluent for silica gel chromatography. R_f_ = 0.34 in EtOAc/Hexanes 80:20 v/v. ^1^H NMR (400 MHz, CDCl_3_, 25 °C, mixture of rotamers): δ = 8.52–8.49 (m, 1H), 7.58 (app td, *J* = 7.7, 1.9 Hz, 1H), 7.17 (app d, *J* = 7.7 Hz, 1H), 7.11–7.07 (m, 1H), 4.30–4.21 (m, 1H), 3.86–3.76 (m, 2H), 2.88–2.79 (m, 2H), 2.34–2.20 (m, 2H), 2.09–1.99 (m, 1H), 1.89–1.80 (m, 1H), 1.42 (s, 9H). ^13^C NMR (100 MHz, CDCl_3_, 25 °C, mixture of rotamers): δ = 161.7, 156.8, 149.3, 136.5, 122.8, 121.2, 79.3, 62.6, 46.6, 35.7, 33.9, 28.6, 22.0. HRMS (ESI-TOF): Calculated for C_15_H_23_N_2_O_2_ [M + H]^+^: 263.1754, found: 263.1766.

### *tert*-Butyl 2-[2-(pyridin-2-yl)ethyl]pyrrolidine-1-carboxylate (3b):

Following general procedure A, compound (±)-**3b** was obtained from *tert*-butyl pyrrolidine-1-carboxylate (102.7 mg, 0.6 mmol, 3 equiv) and 2-vinylpyridine (21.5 μL, 0.2 mmol, 1 equiv) as a colorless oil in 48% yield (26.5 mg). Hexane containing ethyl acetate (25–50%) was used as the eluent for silica gel chromatography. Compound (±)-**3b** is known and the published characterization data matched our own in all respects.^53^ R_f_ = 0.22 in EtOAc/Hexanes 50:50 v/v. ^1^H NMR (400 MHz, CDCl_3_, 25 °C, mixture of rotamers): δ = 8.52–8.46 (m, 1H), 7.61–7.53 (m, 1H), 7.21–7.04 (m 2H), 3.95–3.72 (m, 1H), 3.45–3.23 (m, 2H), 3.88–2.68 (m, 2H), 2.24–2.01 (m, 1H), 1.98–1.66 (m, 5H), 1.42 (s, 9H). ^13^C NMR (100 MHz, CDCl_3_, 25 °C, mixture of rotamers): δ = 162.0, 154.8, 149.4, 136.5, 122.7, 121.1, 79.1, 57.0, 47.9, 46.2, 35.9, 34.9, 34.3, 30.8, 30.1, 28.7, 23.9, 23.2. HRMS (ESI-TOF): Calculated for C_16_H_25_N_2_O_2_ [M + H]^+^: 277.1911, found: 277.1923.

### *tert*-Butyl 2-[2-(pyridin-2-yl)ethyl]piperidine-1-carboxylate (3c):

Following general procedure A, compound (±)-**3c** was obtained from *tert*-butyl piperidine-1-carboxylate (111.2 mg, 0.6 mmol, 3 equiv) and 2-vinylpyridine (21.5 μL, 0.2 mmol, 1 equiv) as a colorless oil in 70% yield (40.7 mg). Hexane containing ethyl acetate (25–50%) was used as the eluent for silica gel chromatography. Compound (±)-**3c** is known and the published characterization data matched our own in all respects.^62^ R_f_ = 0.37 in EtOAc/Hexanes 50:50v/v. ^1^H NMR (400 MHz, CDCl_3_, 25 °C, mixture of rotamers): δ = 8.51–8.48 (m, 1H), 7.55 (app td, *J* = 7.7, 1.9 Hz, 1H), 7.12 (app d, *J* = 7.8 Hz, 1H), 7.07 (ddd, *J* = 7.7, 7.4, 1.2 Hz, 1H), 4.34–4.25 (m, 1H), 4.02–3.91 (m, 1H), 2.84–2.62 (m, 3H), 2.19–2.07 (m, 1H), 1.87–1.75 (m, 1H), 1.68–1.52 (m, 5H), 1.44–1.39 (s, 10H). ^13^C NMR (100 MHz, CDCl_3_, 25 °C, mixture of rotamers): δ = 161.5, 154.8, 149.4, 136.5, 122.9, 121.2, 80.0, 69.3, 67.1, 51.3, 40.0, 35.0, 28.9, 28.5. HRMS (ESI-TOF): Calculated for C_17_H_27_N_2_O_2_ [M + H]^+^: 291.2067, found: 291.2075.

### *tert*-Butyl 2-[2-(pyridin-2-yl)ethyl]azepane-1-carboxylate (3d):

Following general procedure A, compound (±)-**3d** was obtained from *tert*-butyl azepane-1-carboxylate (119.6 mg, 0.6 mmol, 3 equiv) and 2-vinylpyridine (21.5 μL, 0.2 mmol, 1 equiv) as a colorless oil in 41% yield (25.0 mg). Hexane containing ethyl acetate (10–30%) was used as the eluent for silica gel chromatography. R_f_ = 0.33 in EtOAc/Hexanes 30:70v/v. ^1^H NMR (400 MHz, CDCl_3_, 25 °C, mixture of rotamers): δ = 8.53–8.45 (m, 1H), 7.60–7.50 (m, 1H), 7.19–7.03 (m, 2H), 4.24–3.93 (m, 1H), 3.76–3.51 (m, 1H), 2.85–2.63 (m, 3H), 2.15–2.03 (m, 1H), 1.86–1.69 (m, 4H), 1.65–1.54 (m, 2H), 1.46–1.41 (m, 9H), 1.28–1.14 (m, 3H). ^13^C NMR (100 MHz, CDCl_3_, 25 °C, mixture of rotamers): δ = 162.3, 162.0, 156.3, 155.9, 149.3, 149.2, 136.4, 136.4, 123.1, 122.8, 121.1, 121.0, 79.2, 78.9, 55.3, 54.3, 41.9, 41.6, 35.4, 35.3, 35.2, 35.0, 34.9, 34.7, 30.0, 29.0, 28.7, 28.4, 25.2, 24.9. HRMS (ESI-TOF): Calculated for C_18_H_29_N_2_O_2_ [M + H]^+^: 305.2224, found: 305.2229.

### *tert*-Butyl 3-[2-(pyridin-2-yl)ethyl]morpholine-4-carboxylate (3e):

Following general procedure A, compound (±)-**3e** was obtained from *tert*-butyl morpholine-4-carboxylate (112.3 mg, 0.6 mmol, 3 equiv) and 2-vinylpyridine (21.5 μL, 0.2 mmol, 1 equiv) as a colorless oil in 44% yield (25.7 mg). Hexane containing ethyl acetate (35–80%) was used as the eluent for silica gel chromatography. R_f_ = 0.33 in EtOAc/Hexanes 80:20 v/v. ^1^H NMR (400 MHz, CDCl_3_, 25 °C, mixture of rotamers): δ = 8.52–8.49 (m, 1H), 7.57 (app td, *J* = 7.7, 1.8 Hz, 1H), 7.15 (app d, *J* = 7.8 Hz, 1H), 7.10–7.06 (m, 1H), 4.07–3.95 (m, 1H), 3.85–3.72 (m, 3H), 3.55 (dd, *J* = 11.6, 3.2 Hz, 1H), 3.43 (app td, *J* = 11.8, 2.8 Hz, 1H), 3.21–3.10 (m, 1H), 2.87–2.78 (m, 1H), 2.76–2.68 (m, 1H), 2.27–2.17 (m, 1H), 2.11–2.01 (m, 1H), 1.44–1.40 (m, 9H). ^13^C NMR (100 MHz, CDCl_3_, 25 °C, mixture of rotamers): δ = 161.5, 154.8, 149.4, 136.5, 122.9, 121.2, 80.0, 69.3, 67.1, 51.9, 39.8, 35.0, 28.9, 28.5. HRMS (ESI-TOF): Calculated for C_16_H_25_N_2_O_3_ [M + H]^+^: 293.1860, found: 293.1865.

### *tert*-Butyl (2R*,4S*)-4-methyl-2-[2-(pyridin-2-yl)ethyl]piperidine-1-carboxylate (3f):

Following general procedure A, compound (±)-**3f** was obtained from *tert*-butyl 4-methylpiperidine-1-carboxylate (119.6 mg, 0.6 mmol, 3 equiv) and 2-vinylpyridine (21.5 μL, 0.2 mmol, 1 equiv) as a colorless oil in 77% yield (46.9 mg) and > 20:1 diastereomeric ratio. Hexane containing ethyl acetate (25–50%) was used as the eluent for silica gel chromatography. R_f_ = 0.45 in EtOAc/Hexanes 50:50 v/v. ^1^H NMR (400 MHz, CDCl_3_, 25 °C, mixture of rotamers): δ = 8.51–8.47 (m, 1H), 7.55 (app t, *J* = 7.6 Hz, 1H), 7.17–7.02 (m, 2H), 4.46–4.20 (m, 1H), 4.12–3.83 (m, 1H), 2.90–2.63 (m, 3H), 2.14–2.00 (m, 1H), 1.90–1.66 (m, 2H), 1.62–1.49 (m, 2H), 1.45 (s, 9H), 1.22 (ddd, *J* = 13.0, 13.0, 5.9 Hz, 1H), 1.08–0.92 (m, 1H), 0.85 (d, *J* = 6.5 Hz, 3H). ^13^C NMR (100 MHz, CDCl_3_, 25 °C, mixture of rotamers): δ = 162.1, 161.8, 155.2, 149.4, 136.4, 123.1, 122.9, 121.1, 79.3, 79.1, 51.2, 50.3, 39.5, 38.3, 37.8, 37.4, 35.4, 34.2, 30.7, 28.6, 25.5. HRMS (ESI-TOF): Calculated for C_18_H_29_N_2_O_2_ [M + H]^+^: 305.2224, found: 305.2229. Relative stereochemistry was assigned by analogy to similar compounds synthesized from our group.^38^

### *tert*-Butyl 7-[2-(pyridin-2-yl)ethyl]-1,4-dioxa-8-azaspiro[4.5]decane-8-carboxylate (3g):

Following general procedure A, compound (±)-**3g** was obtained from *tert*-butyl 1,4-dioxa-8-azaspiro[4.5]decane-8-carboxylate (146.0 mg, 0.6 mmol, 3 equiv) and 2-vinylpyridine (21.5 μL, 0.2 mmol, 1 equiv) as a colorless oil in 57% yield (39.7 mg). Hexane containing ethyl acetate (35–70%) was used as the eluent for silica gel chromatography. R_f_ = 0.35 in EtOAc/Hexanes 80:20 v/v. ^1^H NMR (400 MHz, CDCl_3_, 25 °C, mixture of rotamers): δ = 8.51–8.48 (m, 1H), 7.56 (app td, *J* = 7.7, 1.8 Hz, 1H), 7.15–7.12 (m, 1H), 7.07 (ddd, *J* = 7.7, 7.5, 1.1 Hz, 1H), 4.56–4.35 (m, 1H), 4.13–4.01 (m, 1H), 3.96–3.83 (m, 4H), 3.10–2.98 (m, 1H), 2.81–2.65 (m, 2H), 2.34–2.23 (m, 1H), 2.00–1.89 (m, 1H), 1.83 (dd, J = 13.7, 6.7 Hz, 1H), 1.74–1.68 (m, 1H), 1.66–1.59 (m, 2H), 1.42 (s, 9H). ^13^C NMR (100 MHz, CDCl_3_, 25 °C, mixture of rotamers): δ = 161.9, 154.9, 149.3, 136.4, 123.0, 121.1, 107.4, 79.7, 64.8, 63.9, 50.8, 37.3, 36.4, 35.7, 34.8, 31.4, 28.5. HRMS (ESI-TOF): Calculated for C_19_H_29_N_2_O_4_ [M + H]^+^: 349.2122, found: 349.2130.

### *tert*-Butyl (2S*,6S*)-2-methyl-6-[2-(pyridin-2-yl)ethyl]piperidine-1-carboxylate (3h):

Following general procedure A, compound (±)-**3h** was obtained from *tert*-butyl 2-methylpiperidine-1-carboxylate (119.6 mg, 0.6 mmol, 3 equiv) and 2-vinylpyridine (21.5 μL, 0.2 mmol, 1 equiv) as a colorless oil in 60% yield (36.5 mg) and > 10:1 diastereomeric ratio. Hexane containing ethyl acetate (25–50%) was used as the eluent for silica gel chromatography. R_f_ = 0.47 in EtOAc/Hexanes 50:50 v/v. ^1^H NMR (400 MHz, CDCl_3_, 25 °C): δ = 8.52–8.49 (m, 1H), 7.57 (app td, *J* = 7.7, 1.9 Hz, 1H), 7.14 (app d, *J* = 7.7 Hz, 1H), 7.08 (ddd, *J* = 7.7, 7.3, 1.2 Hz, 1H), 4.36–4.28 (m, 1H), 4.23–4.15 (m, 1H), 2.88–2.77 (m, 1H), 2.76–2.67 (m, 1H), 2.01–1.89 (m, 2H), 1.74–1.50 (m, 5H), 1.46–1.41 (s, 10H), 1.18 (d, *J* = 7.1 Hz, 3H). ^13^C NMR (100 MHz, CDCl_3_, 25 °C): δ = 162.1, 155.5, 149.4, 136.5, 122.8, 121.1, 79.7, 50.4, 45.8, 36.7, 35.5, 30.4, 28.6, 27.8, 20.7, 14.3. HRMS (ESI-TOF): Calculated for C_18_H_29_N_2_O_2_ [M + H]^+^: 305.2224, found: 305.2235. Note: Relative stereochemistry was determined from compound **5b** after Boc-deprotection.

### *tert*-Butyl cyclohexyl[3-(pyridin-2-yl)propyl]carbamate (3i):

Following general procedure A, compound **3i** was obtained from *tert*-butyl cyclohexyl(methyl)carbamate (128.0 mg, 0.6 mmol, 3 equiv) and 2-vinylpyridine (21.5 μL, 0.2 mmol, 1 equiv) as a light yellow oil in 23% yield (14.6 mg). Hexane containing ethyl acetate (0–20%) was used as the eluent for silica gel chromatography. R_f_ = 0.17 in EtOAc/Hexanes 25:75 v/v. ^1^H NMR (400 MHz, CDCl_3_, 25 °C, mixture of rotamers): δ = 8.53–8.49 (m, 1H), 7.58 (app td, *J* = 7.7, 1.6 Hz, 1H), 7.18–7.06 (m, 2H), 3.93–3.43 (m 1H), 3.24–3.01 (m, 2H), 2.75 (t, *J* = 7.8 Hz, 2H), 1.97–1.85 (m, 2H), 1.80–1.65 (m, 4H), 1.63–1.55 (m, 1H), 1.47–1.38 (m, 10H), 1.35–1.25 (m, 3H), 1.09–0.97 (m, 1H). ^13^C NMR (100 MHz, CDCl_3_, 25 °C, mixture of rotamers): δ = 161.8, 155.6, 149.4, 136.5, 122.8, 121.2, 79.2, 55.2, 43.0, 36.2, 31.5, 31.2, 28.6, 26.2, 25.7. HRMS (ESI-TOF): Calculated for C_19_H_31_N_2_O_2_ [M + H]^+^: 319.2380, found: 319.2397.

### *tert*-Butyl 2-[2-(pyridin-4-yl)ethyl]piperidine-1-carboxylate (3j):

Following general procedure B, compound (±)-**3j** was obtained from *tert*-butyl piperidine-1-carboxylate (111.1 mg, 0.6 mmol, 3 equiv) and 4-vinylpyridine (21.5 μL, 0.2 mmol, 1 equiv) as a colorless oil in 47% yield (27.3 mg). Hexane containing ethyl acetate (25–50%) was used as the eluent for silica gel chromatography. R_f_ = 0.25 in EtOAc/Hexanes 50:50 v/v. ^1^H NMR (400 MHz, CDCl_3_, 25 °C, mixture of rotamers): δ = 8.50–8.44 (m, 2H), 7.11–7.08 (m, 2H), 4.33–4.21 (m, 1H), 4.04–3.93 (m, 1H), 2.79–2.70 (m, 1H), 2.63–2.45 (m, 2H), 2.08–1.95 (m, 1H), 1.73–1.51 (m, 6H), 1.47–1.34 (m, 10H). ^13^C NMR (100 MHz, CDCl_3_, 25 °C, mixture of rotamers): δ = 155.2, 151.2, 149.8, 123.9, 79.4, 50.2, 38.98, 32.2, 30.7, 28.7, 28.6, 25.7, 19.2. HRMS (ESI-TOF): Calculated for C_17_H_27_N_2_O_2_ [M + H]^+^: 291.2067, found: 291.2073.

### *tert*-Butyl 2-[2-(pyridin-3-yl)ethyl]piperidine-1-carboxylate (3k):

Following general procedure B, compound (±)-**3k** was obtained from *tert*-butyl piperidine-1-carboxylate (111.1 mg, 0.6 mmol, 3 equiv) and 3-vinylpyridine (5 M stock solution in MeCN, 40 μL, 0.2 mmol, 1 equiv) as a colorless oil in 27% yield (15.7 mg). Hexane containing ethyl acetate (30–50%) was used as the eluent for silica gel chromatography. R_f_ = 0.17 in EtOAc/Hexanes 50:50 v/v. ^1^H NMR (400 MHz, CDCl_3_, 25 °C, mixture of rotamers): δ = 8.57–8.41 (m, 2H), 7.52–7.48 (m, 1H), 7.20 (dd, *J* = 7.7, 4.8 Hz, 1H), 4.34–4.22 (m, 1H), 4.07–3.95 (s, 1H), 3.82–2.72 (m, 1H), 2.64–2.47 (m, 2H), 2.07–1.89 (m, 2H), 1.73–1.54 (m, 6H), 1.43 (s, 9H). ^13^C NMR (100 MHz, CDCl_3_, 25 °C, mixture of rotamers): δ = 155.3, 150.0, 147.5, 137.5, 135.9, 123.4, 79.4, 50.2, 38.8, 31.6, 30.0, 28.6, 28.3, 25.7, 19.2. HRMS (ESI-TOF): Calculated for C_17_H_27_N_2_O_2_ [M + H]^+^: 291.2067, found: 291.2073.

### *tert*-Butyl 2-[2-(6-bromopyridin-2-yl)ethyl]piperidine-1-carboxylate (3l):

Following general procedure B, compound (±)-**3l** was obtained from *tert*-butyl piperidine-1-carboxylate (111.1 mg, 0.6 mmol, 3 equiv) and 2-bromo-6-vinylpyridine (5 M stock solution in MeCN, 40 μL, 0.2 mmol, 1 equiv) as a colorless oil in 32% yield (23.6 mg). Hexane containing ethyl acetate (0–10%) was used as the eluent for silica gel chromatography. R_f_ = 0.31 in EtOAc/Hexanes 85:15 v/v. ^1^H NMR (400 MHz, CDCl_3_, 25 °C, mixture of rotamers): δ = 7.43 (app t, *J* = 7.7 Hz, 1H), 7.29 (d, *J* = 7.7 Hz 1H), 7.11 (d, *J* = 7.7 Hz, 1H), 4.35–4.24 (m, 1H), 4.04–3.91 (m, 1H), 2.83–2.62 (m, 3H), 2.19–2.07 (m, 1H), 1.86–1.75 (m, 1H), 1.61–1.53 (m, 5H), 1.47–1.34 (m, 10H). ^13^C NMR (100 MHz, CDCl_3_, 25 °C, mixture of rotamers): δ = 163.8, 155.2, 141.7, 138.8, 125.5, 121.9, 80.1, 35.1, 30.0, 28.9, 28.6, 25.8, 19.2. HRMS (ESI-TOF): Calculated for C_17_H_26_BrN_2_O_2_ [M + H]^+^: 369.1172, found: 369.1177.

### *tert*-Butyl 2-[2-(6-methoxypyridin-2-yl)ethyl]piperidine-1-carboxylate (3m):

Following general procedure B, compound (±)-**3m** was obtained from *tert*-butyl piperidine-1-carboxylate (111.1 mg, 0.6 mmol, 3 equiv) and 2-methoxy-6-vinylpyridine (5 M stock solution in MeCN, 40 μL, 0.2 mmol, 1 equiv) as a colorless oil in 42% yield (26.9 mg). Hexane containing ethyl acetate (0–10%) was used as the eluent for silica gel chromatography. R_f_ = 0.58 in EtOAc/Hexanes 85:15 v/v. ^1^H NMR (400 MHz, CDCl_3_, 25 °C, mixture of rotamers): δ = 7.45 (dd, *J* = 8.3, 7.2 Hz, 1H), 6.70 (d, *J* = 7.2 Hz, 1H), 6.53 (d, *J* = 8.3 Hz, 1H), 4.35–4.25 (m, 1H), 4.04–3.95 (m, 1H), 3.91 (s, 3H), 2.86–2.76 (m, 1H), 2.71–2.55 (m, 2H), 2.18–2.06 (m, 1H), 1.90–1.80 (m, 1H), 1.64–1.53 (m, 5H), 1.46–1.36 (m, 10H). ^13^C NMR (100 MHz, CDCl_3_, 25 °C, mixture of rotamers): δ = 163.8, 159.8, 155.3, 138.9, 115.3, 107.4, 79.2, 53.3, 50.5, 39.1, 34.8, 29.5, 28.8, 28.6, 25.8, 19.2. HRMS (ESI-TOF): Calculated for C_18_H_29_N_2_O_3_ [M + H]^+^: 321.2173, found: 321.2180.

### *tert*-Butyl 2-[2-(*1H*-imidazol-2-yl)ethyl]piperidine-1-carboxylate (3n):

Following general procedure C, compound (±)-**3n** was obtained from *tert*-butyl piperidine-1-carboxylate (111.1 mg, 0.6 mmol, 3 equiv) and 2-vinyl-*1H*-imidazole (18.8 mg, 0.2 mmol, 1 equiv) as a light yellow oil in 48% yield (26.8 mg). Ethyl acetate containing isopropyl amine (0–1%) was used as the eluent for silica gel chromatography. R_f_ = 0.22 in EtOAc/MeOH/IPA 90:9:1 v/v. ^1^H NMR (400 MHz, CDCl_3_, 25 °C, mixture of rotamers): δ = 6.94 (s, 2H), 4.34–4.26 (m, 1H), 4.02–3.93 (m, 1H), 2.93–2.73 (m, 2H), 2.46–2.34 (m, 1H), 2.29–2.17 (m, 1H), 1.69–1.54 (m, 4H), 1.52–1.42 (m, 12H). ^13^C NMR (100 MHz, CDCl_3_, 25 °C, mixture of rotamers): δ = 156.7, 148.2, 80.3, 48.4, 39.5, 29.4, 28.60, 28.56, 25.8, 24.4, 19.2. HRMS (ESI-TOF): Calculated for C_15_H_26_N_3_O_2_ [M + H]^+^: 280.2020, found: 280.2034.

### *tert*-Butyl cyclohexyl[3-phenyl-3-(pyridin-2-yl)propyl]carbamate (3o):

Following general procedure B, compound (±)-**3o** was obtained from *tert*-butyl cyclohexyl(methyl)carbamate (128.0 mg, 0.6 mmol, 3 equiv) and 2-(1-phenylvinyl)pyridine (5 M stock solution in MeCN, 40 μL, 0.2 mmol, 1 equiv) as a colorless oil in 59% yield (46.7 mg). Hexane containing ethyl acetate (5–15%) was used as the eluent for silica gel chromatography. R_f_ = 0.42 in EtOAc/Hexanes 25:75 v/v. ^1^H NMR (400 MHz, CDCl_3_, 25 °C, mixture of rotamers): δ = 8.61–8.55 (m, 1H), 7.57 (app t, *J* = 7.7 Hz, 1H), 7.39–7.28 (m, 4H), 7.24–7.15 (m, 2H), 7.15–7.09 (m, 1H), 4.10–3.81 (m, 2H), 3.11–2.91 (m, 2H), 2.54–2.41 (m, 1H), 2.37–2.26 (m, 1H), 1.79–1.65 (m, 5H), 1.63–1.57 (m, 1H), 1.44 (s, 9H), 1.33–1.24 (m, 3H), 1.07–0.96 (m, 1H). ^13^C NMR (100 MHz, CDCl_3_, 25 °C, mixture of rotamers): δ= 163.5, 155.6, 149.4, 143.4, 136.6, 128.7, 128.0, 126.6, 122.7, 121.5, 79.2, 55.2, 52.2, 42.2, 35.8, 31.4, 28.7, 26.1, 25.7. HRMS (ESI-TOF): Calculated for C_25_H_35_N_2_O_2_ [M + H]^+^: 395.2693, found: 395.2698.

### 2-{2-[(2R*,4S*)-4-Methylpiperidin-2-yl]ethyl}pyridine (5a):

To a 25 mL round bottom flask was added a stir bar and (±)-**3f** (63.9 mg, 0.21 mmol, 1 equiv). TFA (2.1 mL) was added, and the reaction was left to stir at room temperature for 16 hours. The TFA was removed via vacuum and 1 M NaOH (10 mL) was added to the crude residue and stirred for 10 minutes. The NaOH was extracted with diethyl ether (4 × 5 mL) and the combined organic layers were dried over Na_2_SO_4_. The dried organic layer was filtered, and the solvent was removed under reduced pressure resulting in a light-yellow oil in 96% yield (41.2 mg) and >20:1 diastereomeric ratio. No further purification was performed. ^1^H NMR (600 MHz, CDCl_3_, 25 °C): δ = 8.50–8.48 (m, 1H), 7.56 (app td, *J* = 7.7, 1.8 Hz, 1H), 7.13 (app d, *J* = 7.7 Hz, 1H), 7.07 (app dd, *J* = 7.6, 7.2 Hz, 1H), 2.87–2.75 (m, 5H), 2.54 (s, 1H), 1.93–1.83 (m, 2H), 1.81–1.74 (m, 1H), 1.67–1.61 (m, 1H), 1.48–1.38 (m, 2H), 1.27–1.20 (m, 1H), 0.93 (d, *J* = 7.0 Hz, 3H). ^13^C NMR (150 MHz, CDCl_3_, 25 °C): δ = 162.1, 149.3, 136.5, 122.8, 121.1, 51.0, 40.8, 38.7, 35.3, 34.9, 33.4, 26.0, 19.9. HRMS (ESI-TOF): Calculated for C_13_H_21_N_2_ [M + H]^+^: 205.1699, found: 205.1715.

### 2-{2-[(2S*,6S*)-6-Methylpiperidin-2-yl]ethyl}pyridine (5b):

To a 25 mL round bottom flask was added a stir bar and (±)-**3h** (57.0 mg, 0.19 mmol, 1 equiv). TFA (1.9 mL) was added, and the reaction was left to stir at room temperature for 16 hours. The TFA was removed via vacuum and 1 M NaOH (10 mL) was added to the crude residue and stirred for 10 minutes. The NaOH was extracted with diethyl ether (4 × 5 mL) and the combined organic layers were dried over Na_2_SO_4_. The dried organic layer was filtered, and the solvent was removed under reduced pressure resulting in a light-yellow oil in 93% yield (36.1 mg) and >10:1 diastereomeric ratio. No further purification was performed. ^1^H NMR (600 MHz, CDCl_3_, 25 °C): δ = 8.50–8.47 (m, 1H), 7.56 (app td, *J* = 7.7, 1.9 Hz, 1H), 7.12 (app d, *J* = 7.7 Hz, 1H), 7.07 (app dd, *J* = 7.7, 7.0 Hz, 1H), 2.82 (t, *J* = 8.0 Hz, 2H), 2.59 (dqd, *J* = 12.6, 6.3, 2.6 Hz, 1H), 2.54 (dtd, *J* = 10.9, 6.4, 2.5 Hz, 1H), 1.83–1.72 (m, 4H), 1.68–1.64 (m, 1H), 1.58–1.54 (m, 1H), 1.30 (app qt, *J* = 13.1, 3.9 Hz 1H), 1.07–0.97 (m, 5H). ^13^C NMR (150 MHz, CDCl_3_, 25 °C): δ = 162.1, 149.3, 136.4, 122.7, 121.1, 56.7, 52.5, 37.5, 35.0, 34.4, 32.2, 24.9, 23.2. HRMS (ESI-TOF): Calculated for C_13_H_21_N_2_ [M + H]^+^: 205.1699, found: 205.1711. Note: Relative stereochemistry was determined from the coupling constants for the α and α’ protons.

## Supplementary Material

SI

Copies of the ^1^H and ^13^C NMR spectra for all title compounds are provided in the supplementary material.

## References

[1] Taylor RD; Maccoss M; Lawson ADG J. Med. Chem 2014, 57, 5845. 10.1021/jm4017625 24471928

[2] Vitaku E; Smith DT; Njardarson JT J. Med. Chem 2014, 57, 10257. 10.1021/jm501100b 25255204

[3] Marshall CM; Federice JG; Bell CN; Cox PB; Njardarson JT J. Med. Chem 2024, 67, 11622. 10.1021/acs.jmedchem.4c01122 38995264

[4] Campos KR Chem. Soc. Rev 2007, 36, 1069. 10.1039/B607547A 17576475

[5] Dutta S; Li B; Rickertsen DRL; Valles DA; Seidel D SynOpen 2021, 05, 173. 10.1055/s-0040-1706051 PMC8612105 34825124

[6] Holmberg-Douglas N; Nicewicz DA Chem. Rev 2022, 122, 1925. 10.1021/acs.chemrev.1c00311 34585909 PMC8939264

[7] Capaldo L; Ravelli D; Fagnoni M Chem. Rev 2022, 122, 1875. 10.1021/acs.chemrev.1c00263 34355884 PMC8796199

[8] Condie AG; González-Gómez JC; Stephenson CRJ J. Am. Chem. Soc 2010, 132, 1464. 10.1021/ja909145y 20070079

[9] Hari DP; König B Org. Lett 2011, 13, 3852. 10.1021/ol201376v 21744842

[10] McNally A; Prier CK; MacMillan DWC Science 2011, 334, 1114. 10.1126/science.1213920 22116882 PMC3266580

[11] Kohls P; Jadhav D; Pandey G; Reiser O Org. Lett 2012, 14, 672. 10.1021/ol202857t 22260623

[12] Fan X-Z; Rong J-W; Wu H-L; Zhou Q; Deng H-P; Tan JD; Xue C-W; Wu L-Z; Tao H-R; Wu J Angew. Chem., Int. Ed 2018, 57, 8514. 10.1002/anie.201803220 29718584

[13] Papadopoulos GN; Kokotou MG; Spiliopoulou N; Nikitas NF; Voutyritsa E; Tzaras DI; Kaplaneris N; Kokotos CG ChemSusChem 2020, 12, 5934. 10.1002/cssc.202001892 32833347

[14] Angioni S; Ravelli D; Emma D; Dondi D; Fagnoni M; Albini A Adv. Synth. Catal 2008, 350, 2209. 10.1002/adsc.200800378

[15] Lipp A; Lahm G; Opatz T J. Org. Chem 2016, 81, 4890. 10.1021/acs.joc.6b00715 27128627

[16] Shaw MH; Shurtleff VW; Terrett JA; Cuthbertson JD; MacMillan DWC Science 2016, 352, 1304. 10.1126/science.aaf6635 27127237 PMC5114852

[17] Choi GJ; Zhu Q; Miller DC; Gu CJ; Knowles RR Nature 2016, 539, 268. 10.1038/nature19811 27732585 PMC5704892

[18] Le C; Liang Y; Evans RW; Li X; MacMillan DWC Nature 2017, 547, 79. 10.1038/nature22813 28636596 PMC5655994

[19] Xu S; Chen H; Zhou Z; Kong W Angew. Chem., Int. Ed 2021, 60, 7405. 10.1002/anie.202014632 33300196

[20] Mao E; MacMillan DWC J. Am. Chem. Soc 2023, 145, 2787. 10.1021/jacs.2c13396 36696091 PMC10680140

[21] McManus JB; Onuska NPR; Nicewicz DA J. Am. Chem. Soc 2018, 140, 9056. 10.1021/jacs.8b04890 29986129

[22] McManus JB; Onuska NPR; Jeffreys MS; Goodwin NC; Nicewicz D Org. Lett 2020, 22, 679. 10.1021/acs.orglett.9b04456 31904980

[23] Kanegusuku ALG; Roizen JL Angew. Chem., Int. Ed 2021, 60, 21116. 10.1002/anie.202016666 PMC8382814 33629454

[24] Romero NA; Nicewicz DA 2016, 116, 10075. 10.1021/acs.chemrev.6b00057 27285582

[25] Zilate B; Fischer C; Sparr C Chem. Commun 2020, 56, 1767. 10.1039/C9CC08524F 31998897

[26] Tlili A; Lakhdar S Angew. Chem., Int. Ed 2021, 60, 19526. 10.1002/anie.202102262 33881207

[27] Nguyen VT; Nguyen VD; Haug GC; Dang HT; Jin S; Li Z; Flores-Hansen C; Benavides BS; Arman HD; Larionov OV ACS Catal 2019, 9, 9485. 10.1021/acscatal.9b02951 35223139 PMC8872120

[28] Dang HT; Haug GC; Nguyen VT; Vuong NTH; Nguyen VD; Arman HD; Larionov OV ACS Catal 2020, 10, 11448. 10.1021/acscatal.0c03440 36636662 PMC9833602

[29] Zhuang K; Haug GC; Wang Y; Yin S; Sun H; Huang S; Trevino R; Shen K; Sun Y; Huang C; Qin B; Liu Y; Cheng M; Larionov OV; Jin S J. Am. Chem. Soc 2024, 146, 8508. 10.1021/jacs.3c14828 38382542

[30] Zubkov MO; Kosobokov MD; Levin VV; Kokorekin VA; Korlyukov AA; Hu J; Dilman AD 2020, 11, 737. 10.1039/C9SC04643G PMC8146146 34123046

[31] Zhilyaev KA; Lipilin DL; Kosobokov MD; Samigullina AI; Dilman AD Adv. Synth. Catal 2022, 364, 3295. 10.1002/adsc.202200515

[32] Kim J; Sun X; van der Worp BA; Ritter T Nat. Catal 2023, 6, 196. 10.1038/s41929-023-00914-7

[33] Laze L; Quevedo-Flores B; Bosque I; Gonzalez-Gomez JC Org. Lett 2023, 25, 8541. 10.1021/acs.orglett.3c02619 37819209 PMC10714401

[34] Toriumi N; Inoue T; Iwasawa N J. Am. Chem. Soc 2022, 144, 19592. 10.1021/jacs.2c09318 36219695

[35] Matsuda Y; Nakajima M; Nemoto T ACS Catal 2023, 13, 10224. 10.1021/acscatal.3c01654

[36] Andrews JA; Kalepu J; Palmer CF; Poole DL; Christensen KE; Willis MC J. Am. Chem. Soc 2023, 145, 21623. 10.1021/jacs.3c07974 37738304 PMC10557147

[37] Adili A; Korpusik AB; Seidel D; Sumerlin BS Angew. Chem., Int. Ed 2022, 61, e202209085. 10.1002/anie.202209085 35989222

[38] Schué E; Rickertsen DRL; Korpusik AB; Adili A; Seidel D; Sumerlin BS Chem. Sci 2023, 14, 11228. 10.1039/D3SC03827K 37860640 PMC10583696

[39] Korpusik AB; Adili A; Bhatt K; Anatot JE; Seidel D; Sumerlin BS J. Am. Chem. Soc 2023, 145, 10480. 10.1021/jacs.3c02497 37155970

[40] Bhatt K; Adili A; Tran AH; Elmallah KM; Ghiviriga I; Seidel D J. Am. Chem. Soc 2024, 146, 26331. 10.1021/jacs.4c08754 39263993 PMC11558692

[41] Rickertsen DRL; Crow JD; Das T; Ghiviriga I; Hirschi JS; Seidel D ACS Catal 2024, 14, 14574. 10.1021/acscatal.4c04897 39822273 PMC11735037

[42] Fukuzumi S; Yuasa J; Satoh N; Suenobu T J. Am. Chem. Soc 2004, 126, 7585. 10.1021/ja031649h 15198606

[43] Lasky MR; Liu E-C; Remy MS; Sanford MS J. Am. Chem. Soc 2024, 146, 14799. 10.1021/jacs.4c02991 38759094 PMC11577968

[44] Choi GJ; Knowles RR J. Am. Chem. Soc 2015, 137, 29, 9226. 10.1021/jacs.5b05377 PMC4643263 26166022

[45] Miyazawa K; Yasu Y; Koike T; Akita M Chem. Commun 2013, 49, 7249. 10.1039/C3CC42695E 23842713

[46] Lima F; Sharma UK; Grunenberg L; Saha D; Johannsen S; Sedelmeier J; Van der Eycken EV; Ley SV Angew. Chem., Int. Ed 2017, 56, 15136. 10.1002/anie.201709690 PMC5708277 29024307

[47] Capaldo L; Fagnoni M; Ravelli D Chem. - Eur. J 2017, 23, 6527. 10.1002/chem.201701346 28346716

[48] Lee KN; Lei Z; Ngai M-Y J. Am. Chem. Soc 2017, 139, 5003. 10.1021/jacs.7b01373 28358497 PMC5623169

[49] Cao K; Tan SM; Lee R; Yang S; Jia H; Zhao X; Qiao B; Jiang Z J. Am. Chem. Soc 2019, 141, 5437. 10.1021/jacs.9b00286 30866625

[50] Yin Y; Dai Y; Jia H; Li J; Bu L; Qiao B; Zhao X; Jiang Z J. Am. Chem. Soc 2018, 140, 6083. 10.1021/jacs.8b01575 29634250

[51] Z Y-L; Wang G-H; Wu Y; Zhu C-Y; Wang P Org. Lett 2021, 23, 8522. 10.1021/acs.orglett.1c03229 34662135

[52] Tan Y; Yin Y; Cao S; Zhao X; Qu G; Jiang Z; Chinese J. Catal 2022, 43, 558. 10.1016/S1872-2067(21)63887-1

[53] Lovett GH; Sparling BA Org. Lett 2016, 18, 3494. 10.1021/acs.orglett.6b01712 27364853

[54] Schwarz J; König B Green Chem 2016, 18, 4743. 10.1039/C6GC01101B

[55] Quan Y; Song Y; Shi W; Xu Z; Chen JS; Jiamg X; Wamg C; Lin W J. Am. Chem. Soc 2020, 142, 8602. 10.1021/jacs.0c02966 32336088

[56] Ranjan P; Pillitteri S; Coppola G; Oliva M; Van der Eycken EV; Sharma UK ACS Catal 2021, 11, 10862. 10.1021/acscatal.1c02823 PMC8487034 34632333

[57] Pizzio MG; Mata EG; Dauban P; Saget T Eur. J. Org. Chem 2023, 26, e202300616. 10.1002/ejoc.202300616

[58] Alunni S; Laureti V; Ottavi L; Ruzziconi R J. Org. Chem 2003, 68, 718. 10.1021/jo020603o 12558390

[59] Xiong S-S; Jian C; Mo Y-Q; Hu W; He Y-K; Ren B-Y; Yang Y-M; Li S J. Org. Chem 2024, 89, 10077. 10.1021/acs.joc.4c00929 38937142

[60] Yang H; Wang E; Yang P; Lv H; Zhang X Org. Lett 2017, 19, 5062. 10.1021/acs.orglett.7b02262 28885851

[61] Pu T-L; Wang X-Y; Sun Z-B; Dong X-Y; Wang Q-Y; Zang S-Q Angew. Chem., Int. Ed 2024, 63, e202402363. 10.1002/anie.202402363 38497318

[62] Ruiz A; Mariani E; Protti S; Fagnoni M Org. Chem. Front 2024, 11, 661. 10.1039/D3QO01856C

