# Supplementary material for: Photocatalytic α-alkylation of carbamates with vinyl azaarenes
Source: ARKIVOC. Author manuscript; Available in PMC 2026 Jan 29. (PMC12850520; doi:10.24820/ark.5550190.p012.325)
Supplement: SI [file NIHMS2114817-supplement-SI.pdf]

Supplementary Material

Photocatalytic α-alkylation of carbamates with vinyl azaarenes

Dillon R. L. Rickertsen, Emma N. George, and Daniel Seidel*

Center for Heterocyclic Compounds, Department of Chemistry, University of Florida, Gainesville, Florida 32611, United States
Email: seidel@chem.ufl.edu

Table of Contents

NMR Spectra S2

^{1}H-NMR of (±)-**3a** (400 MHz, $CDCl_3$, 25 °C)

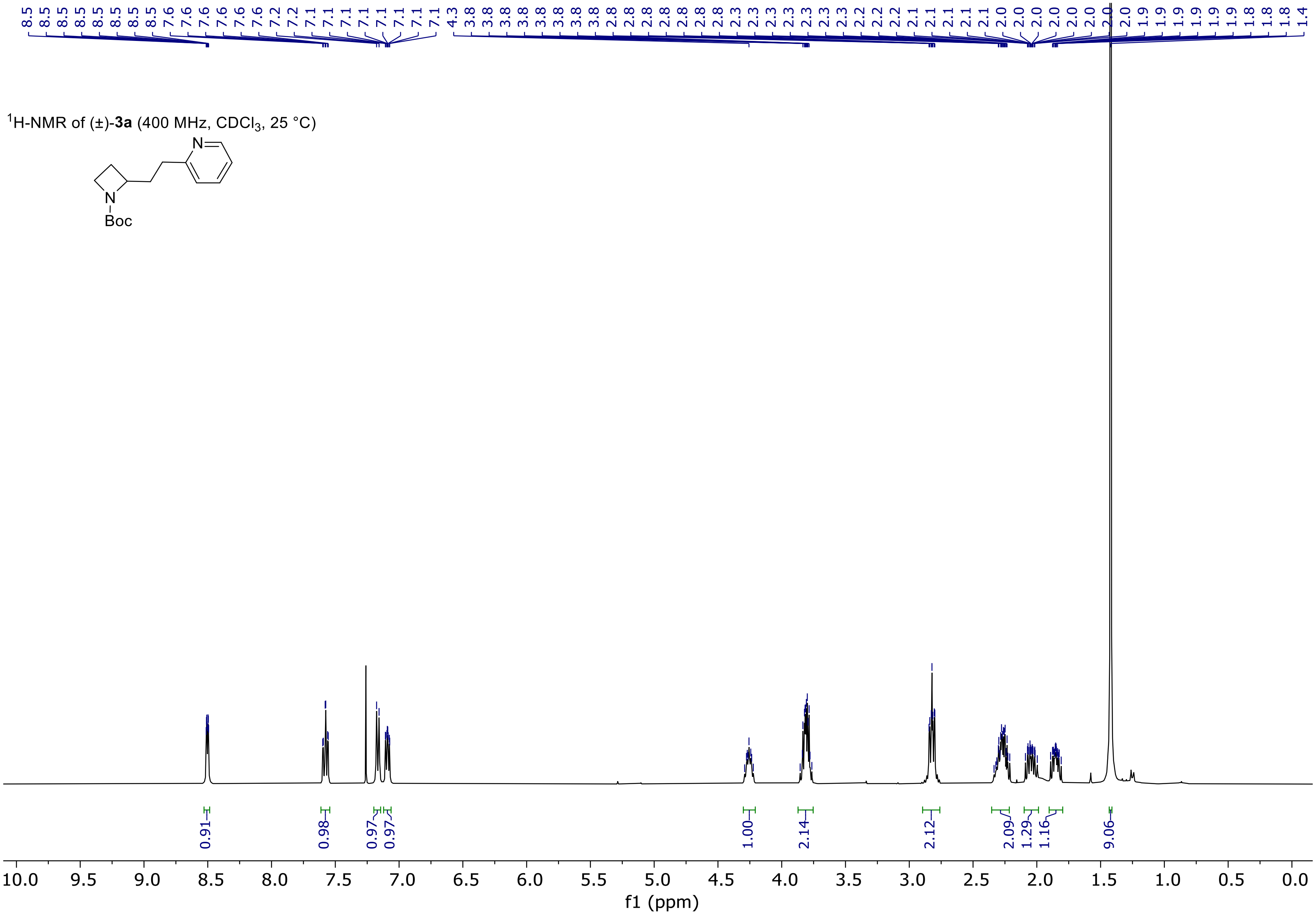

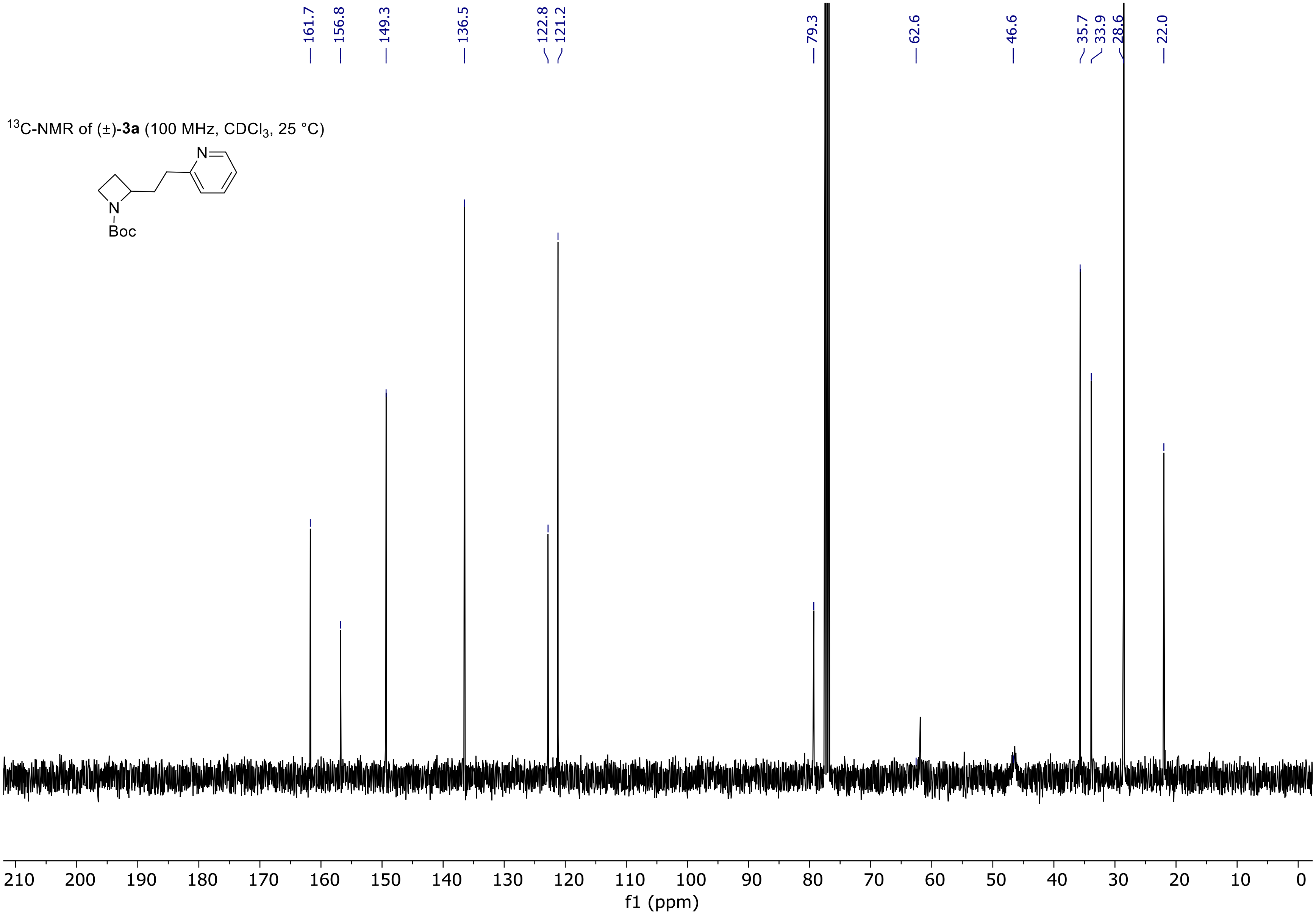
161.7
156.8
149.3
136.5
122.8
121.2
79.3
62.6
46.6
35.7
33.9
28.6
22.0
^{13}C-NMR of (±)-**3a** (100 MHz, $CDCl_3$, 25 °C)
N
N
Boc
210 200 190 180 170 160 150 140 130 120 110 100 90 80 70 60 50 40 30 20 10 0
f1 (ppm)

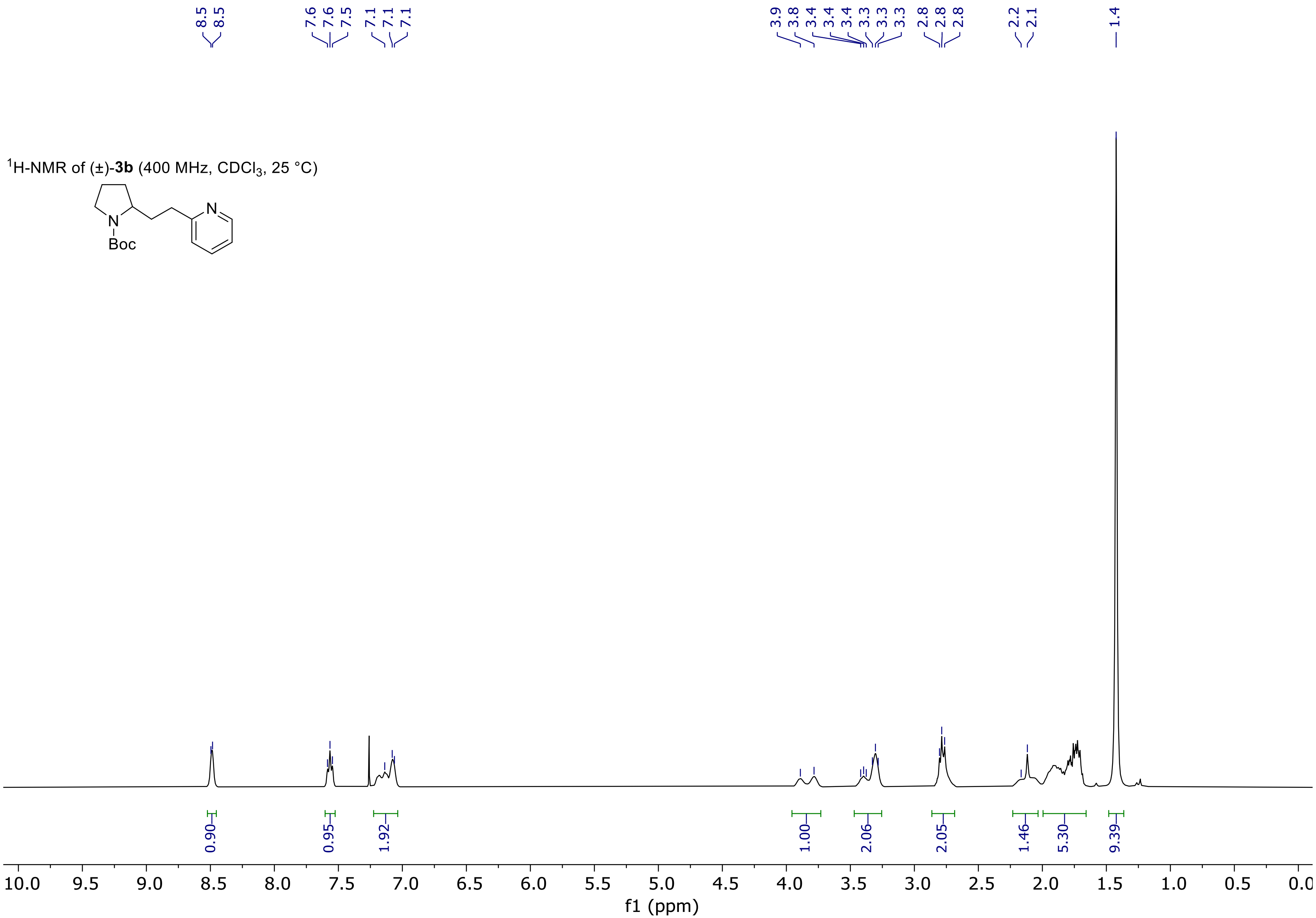

^{1}H-NMR of (±)-**3b** (400 MHz, CDCl3, 25 °C)
N
Boc
N
f1 (ppm)

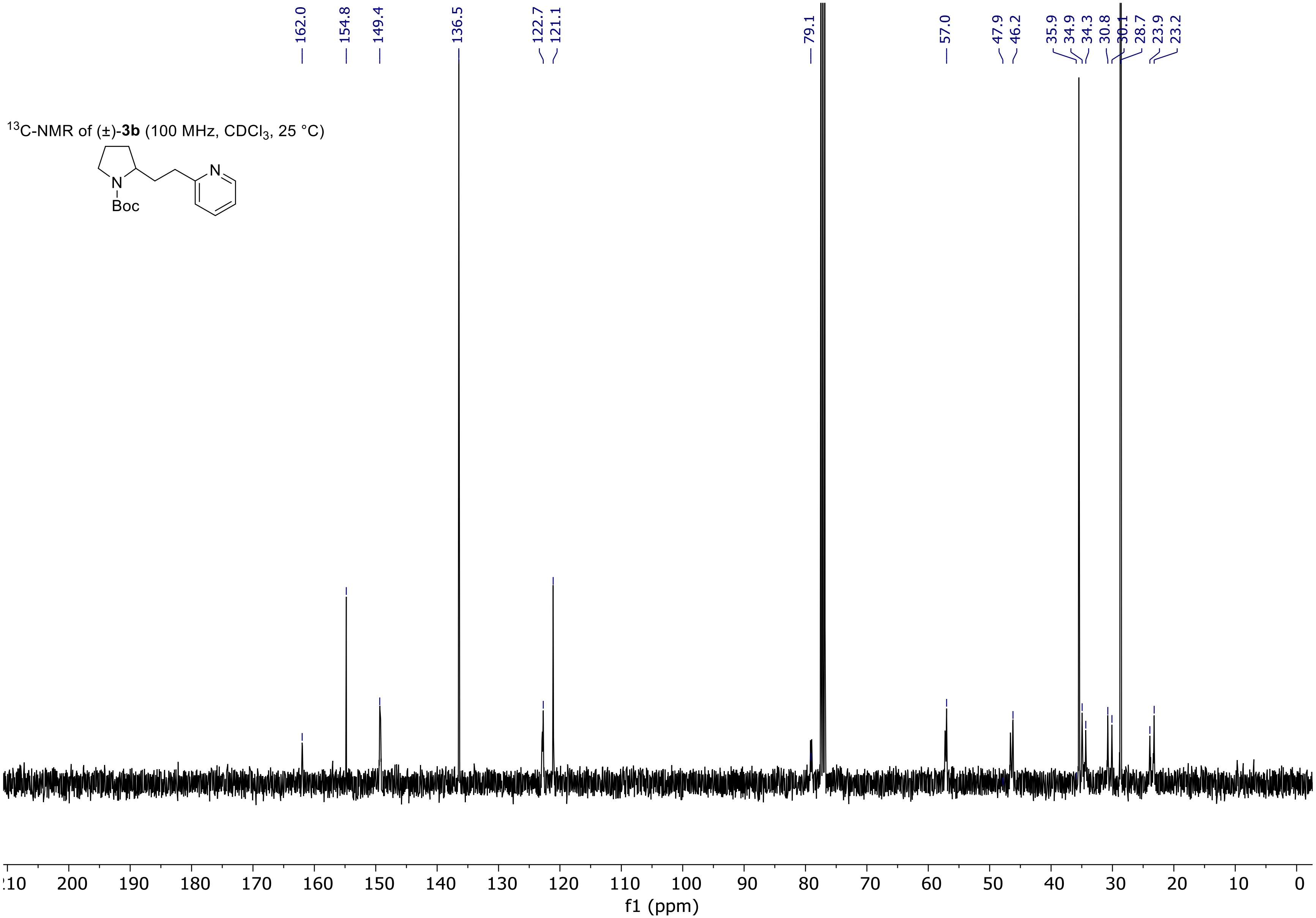
162.0
154.8
149.4
136.5
122.7
121.1
79.1
57.0
47.9
46.2
35.9
34.9
34.3
30.8
30.1
28.7
23.9
23.2
^{13}C-NMR of (±)-**3b** (100 MHz, $CDCl_3$, 25 °C)
N
N
Boc
210 200 190 180 170 160 150 140 130 120 110 100 90 80 70 60 50 40 30 20 10 0
f1 (ppm)

^{1}H-NMR of (±)-**3c** (400 MHz, $CDCl_3$, 25 °C)

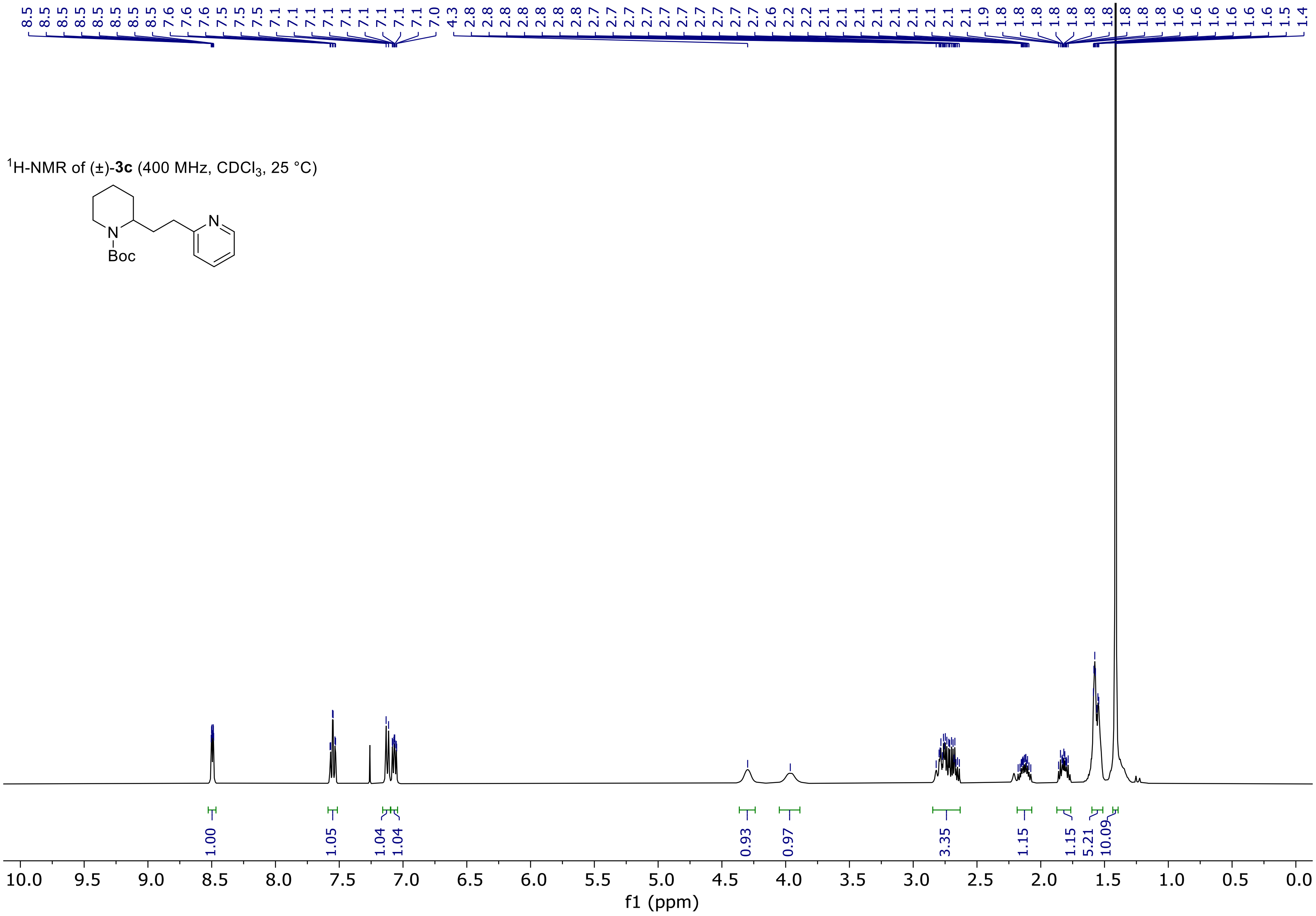

^{13}C-NMR of (±)-**3c** (100 MHz, $CDCl_3$, 25 °C)

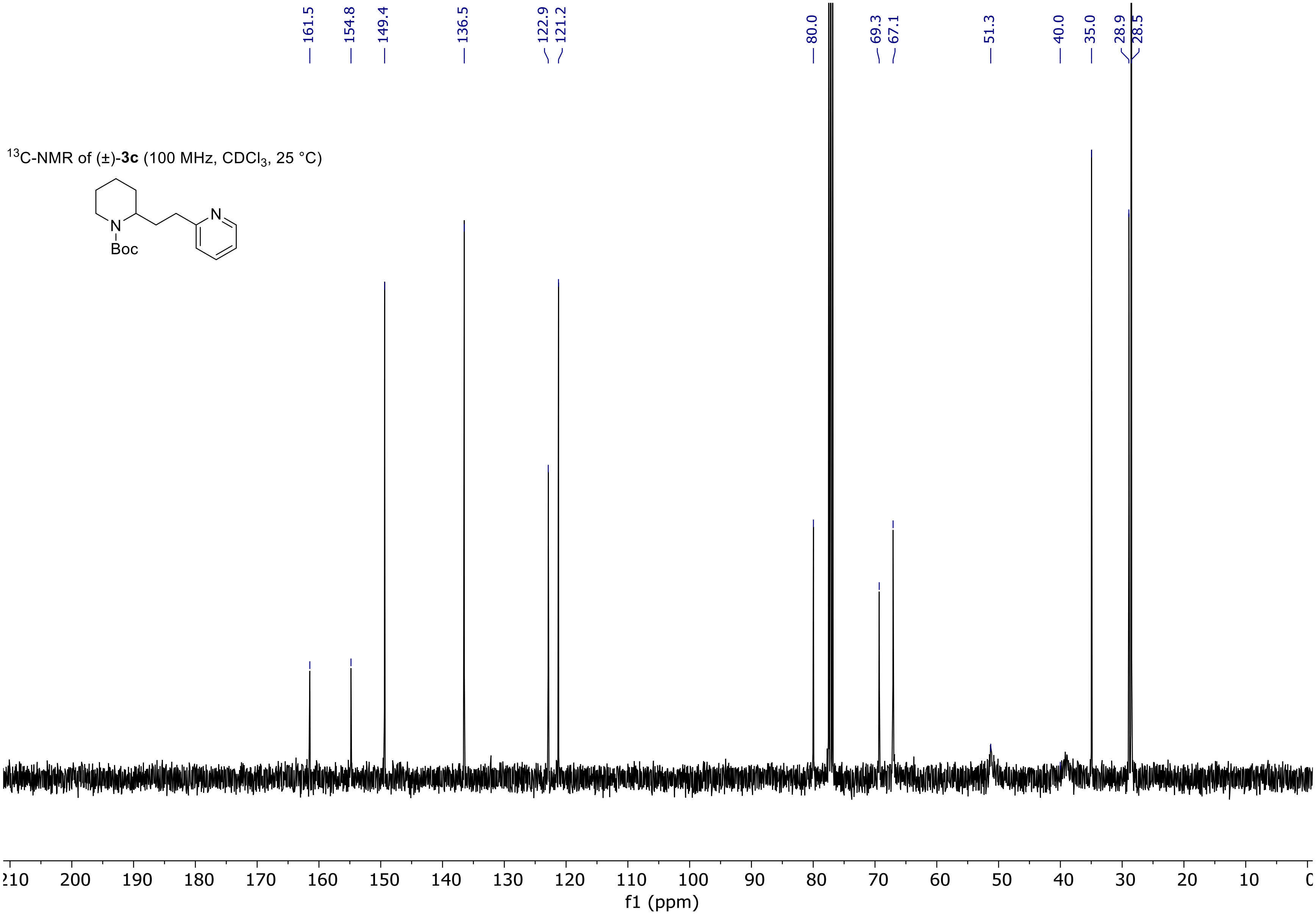

^{1}H-NMR of (±)-**3d** (400 MHz, $CDCl_3$, 25 °C)

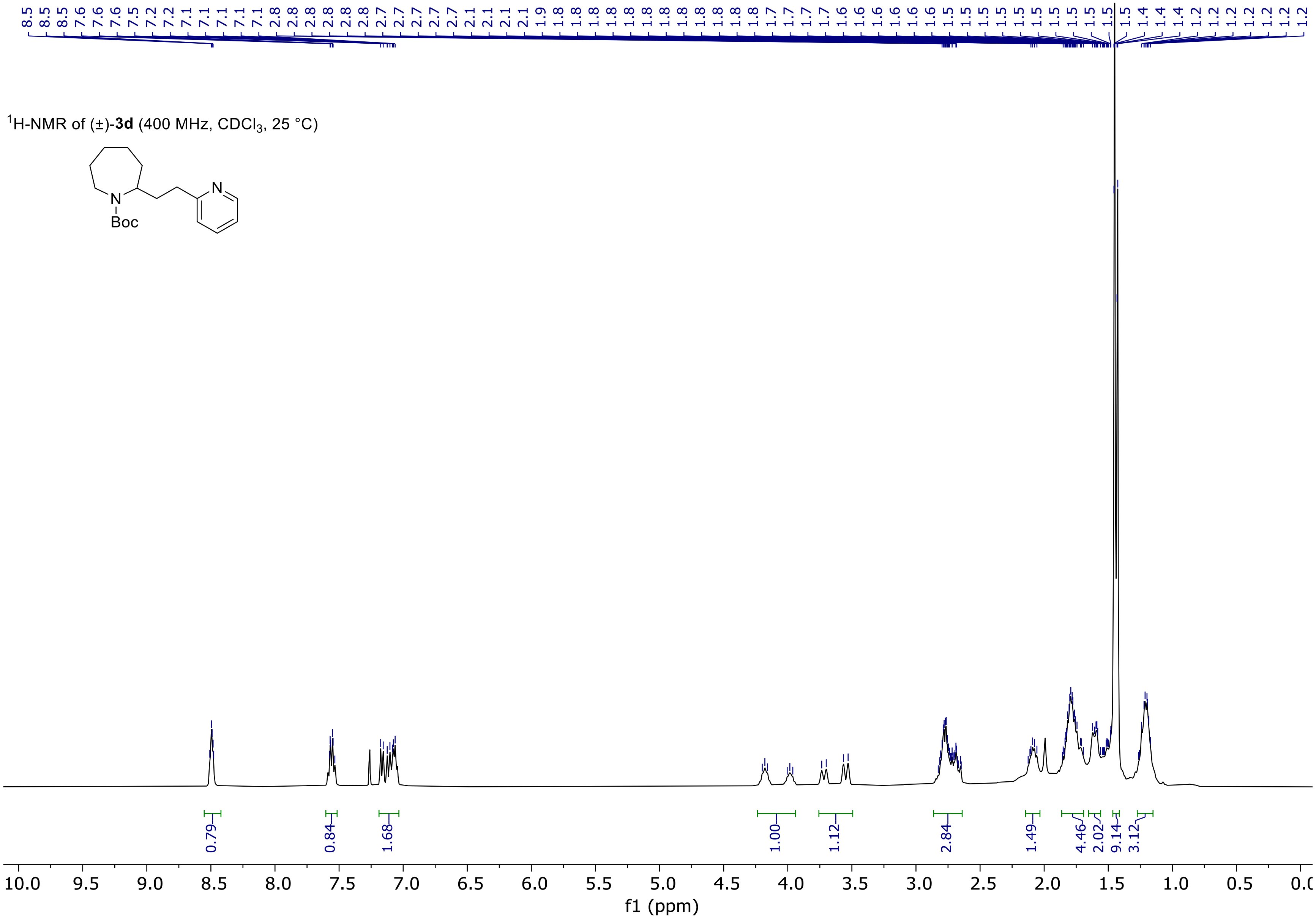

^{13}C-NMR of (±)-**3d** (100 MHz, $CDCl_3$, 25 °C)

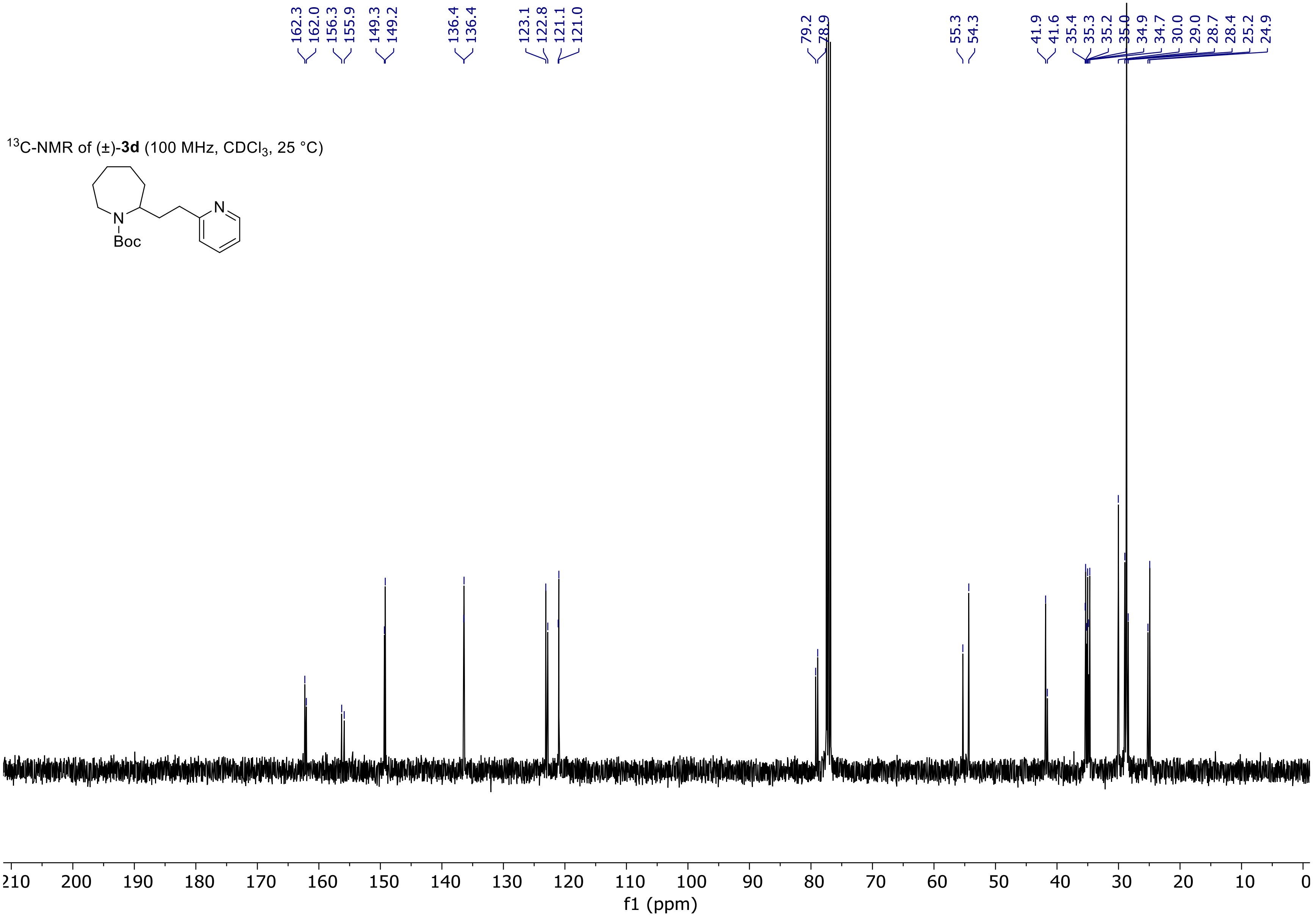

^{1}H-NMR of (±)-**3e** (400 MHz, $CDCl_3$, 25 °C)

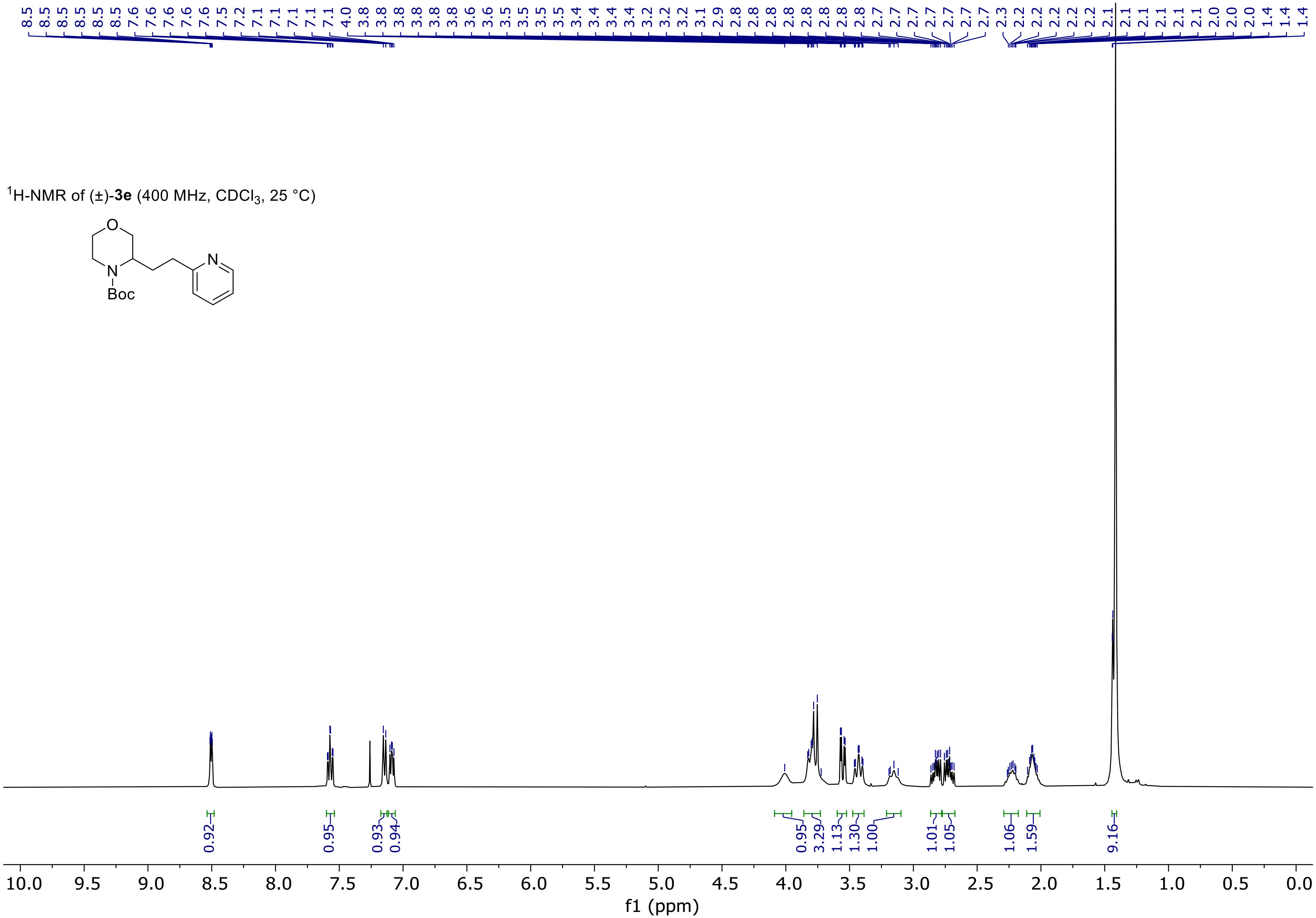

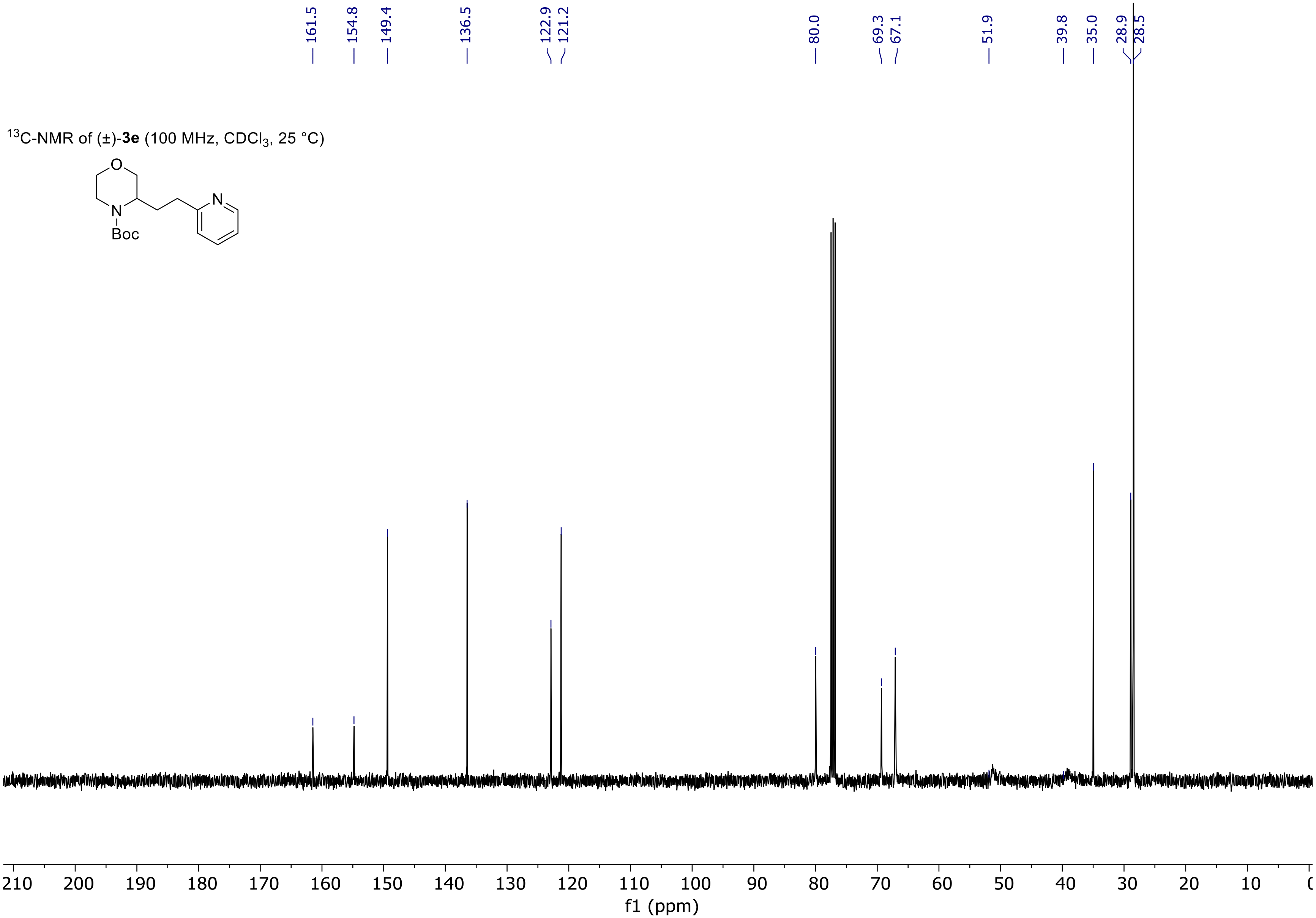
161.5
154.8
149.4
136.5
122.9
121.2
80.0
69.3
67.1
51.9
39.8
35.0
28.9
28.5
^{13}C-NMR of (±)-**3e** (100 MHz, $CDCl_3$, 25 °C)
O
N
N
Boc
210
200
190
180
170
160
150
140
130
120
110
100
90
80
70
60
50
40
30
20
10
f1 (ppm)

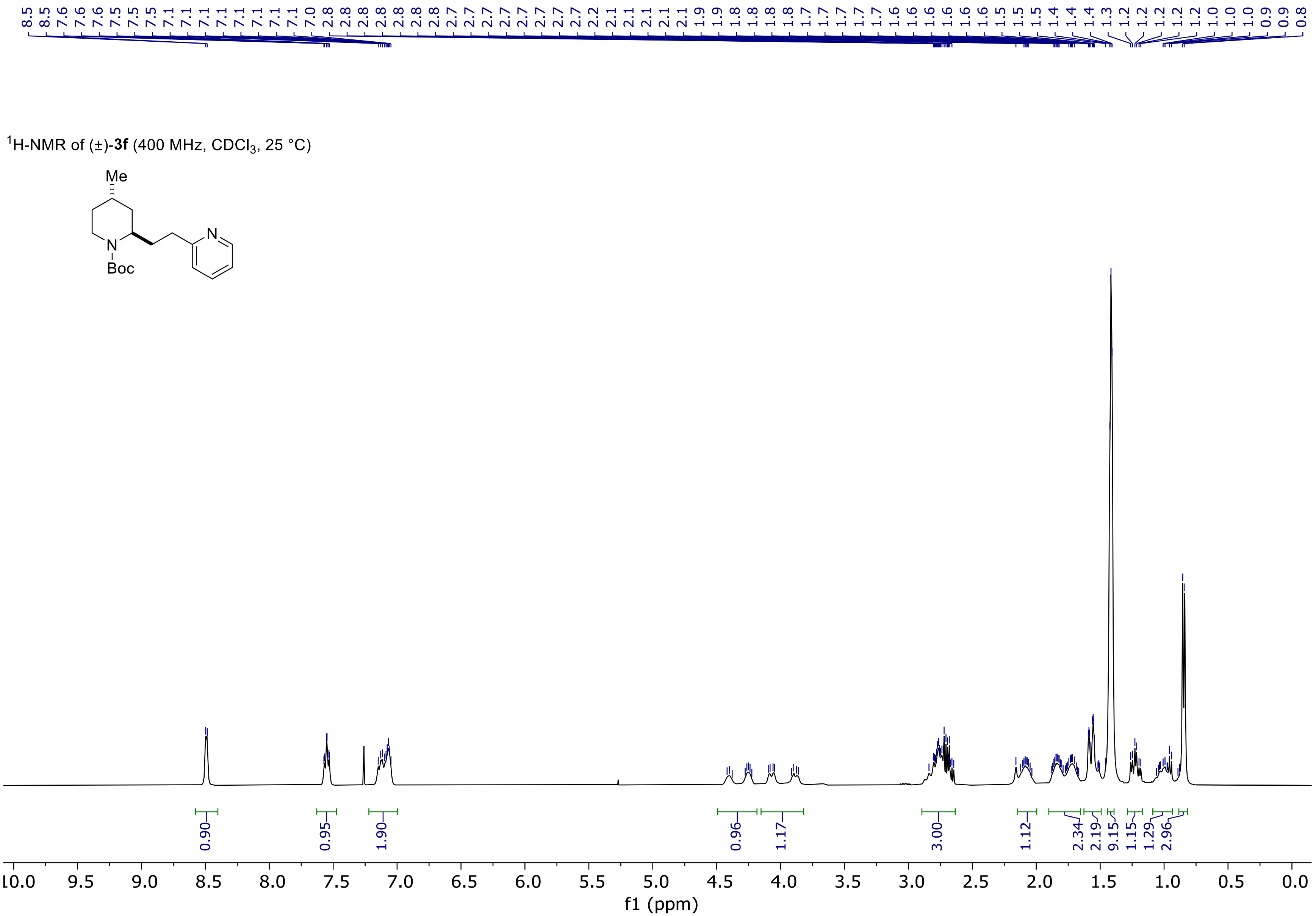
^{1}H-NMR of (±)-**3f** (400 MHz, $CDCl_3$, 25 °C)
Me
N
Boc
N
0.90
0.95
1.90
0.96
1.17
3.00
1.12
2.34
2.19
9.15
1.15
1.29
2.96
f1 (ppm)

^{13}C-NMR of (±)-**3f** (100 MHz, $CDCl_3$, 25 °C)

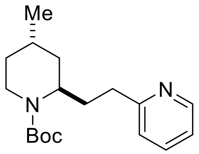

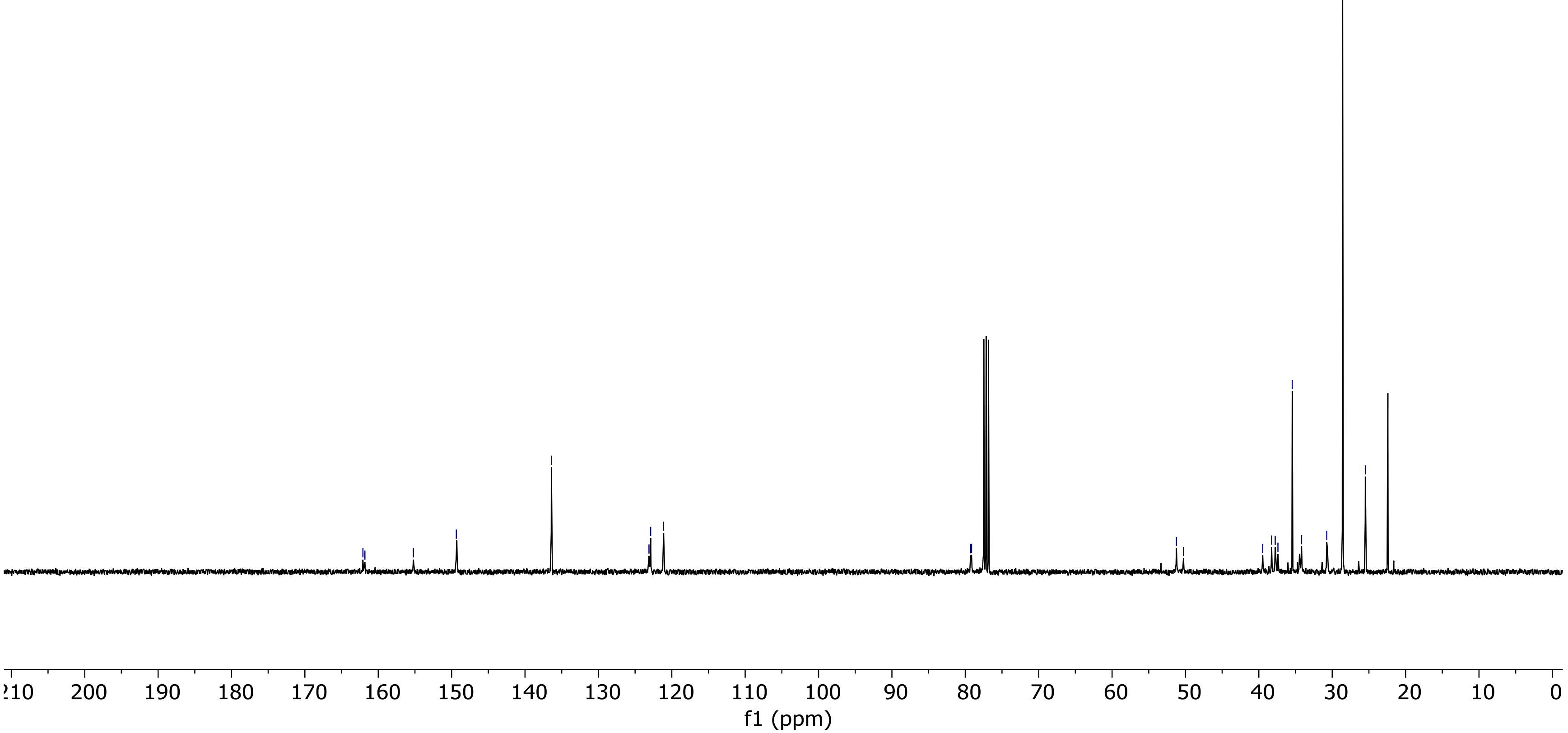

^{1}H-NMR of (±)-**3g** (400 MHz, $CDCl_3$, 25 °C)

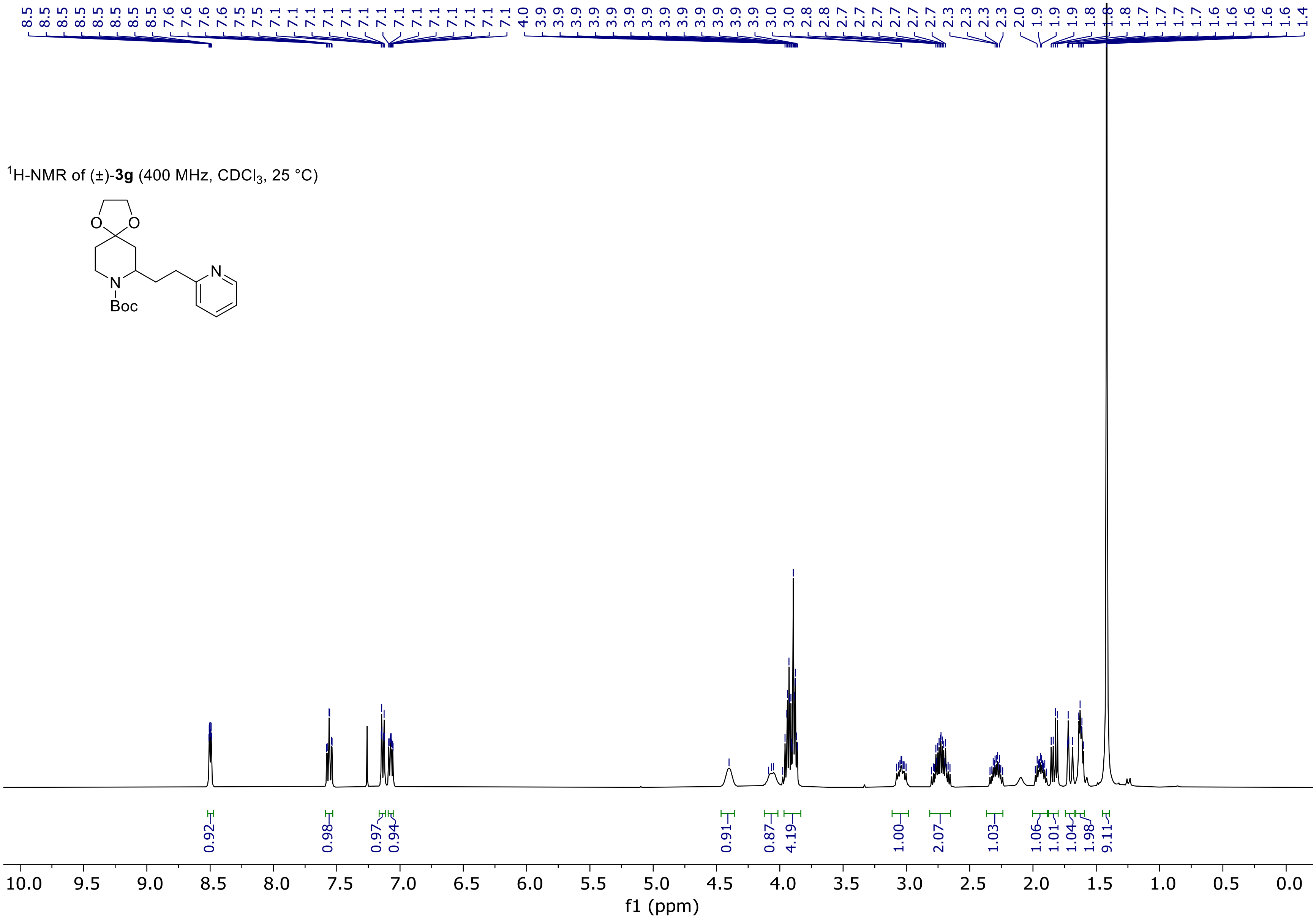

^{13}C-NMR of (±)-**3g** (100 MHz, $CDCl_3$, 25 °C)

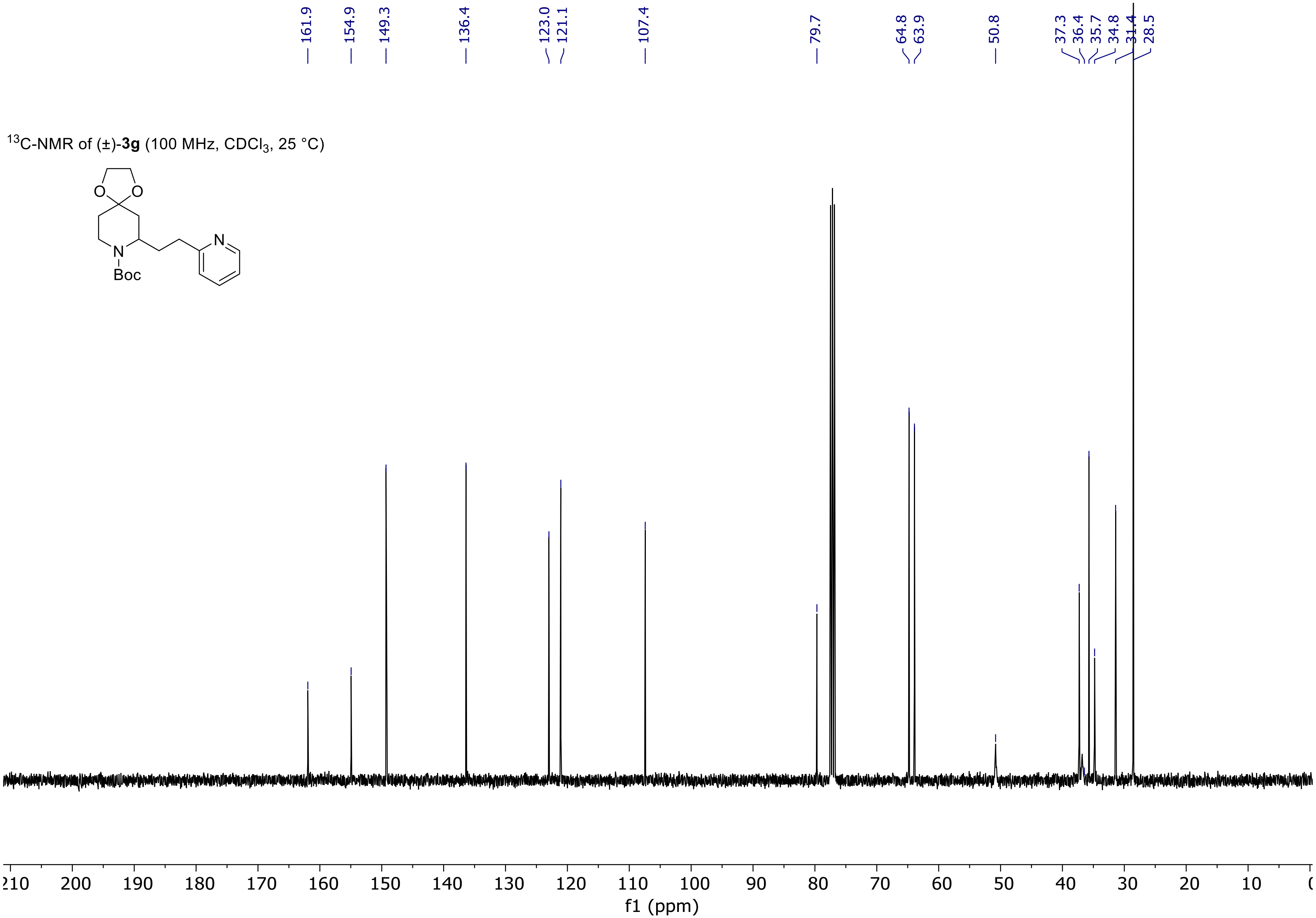

^{1}H-NMR of (±)-**3h** (400 MHz, $CDCl_3$, 25 °C)

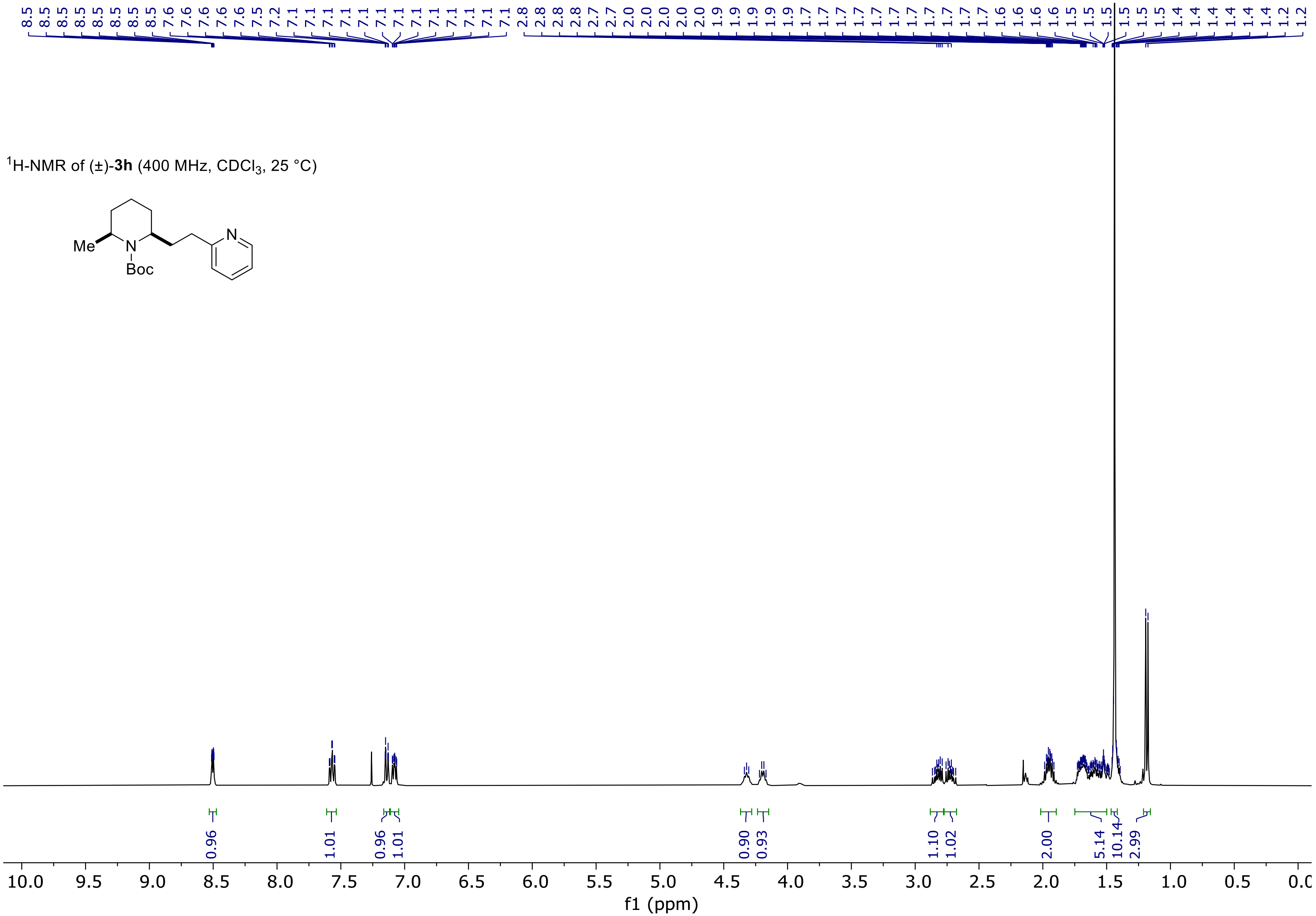

^{13}C-NMR of (±)-**3h** (100 MHz, $CDCl_3$, 25 °C)

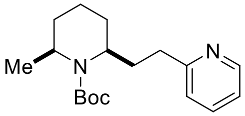

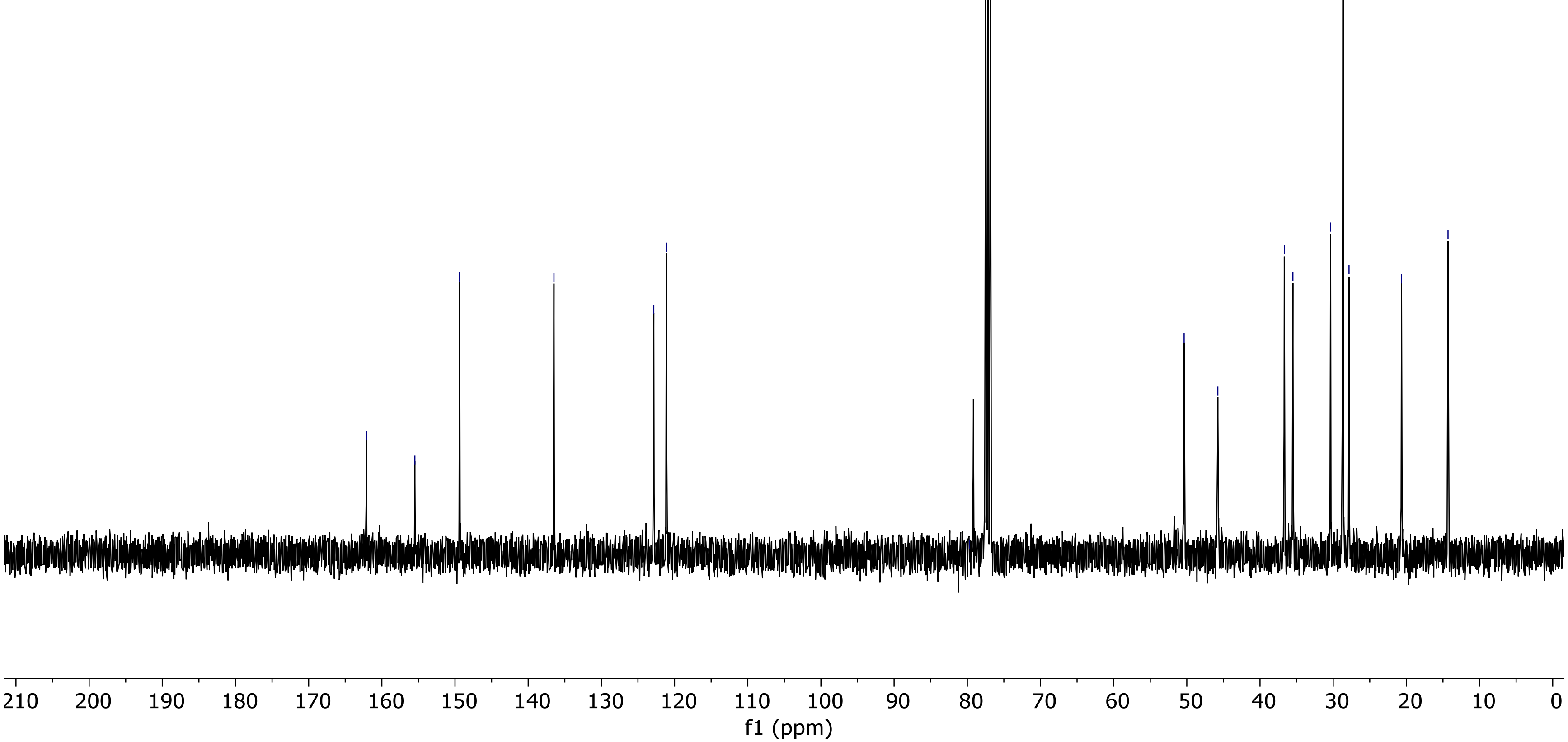

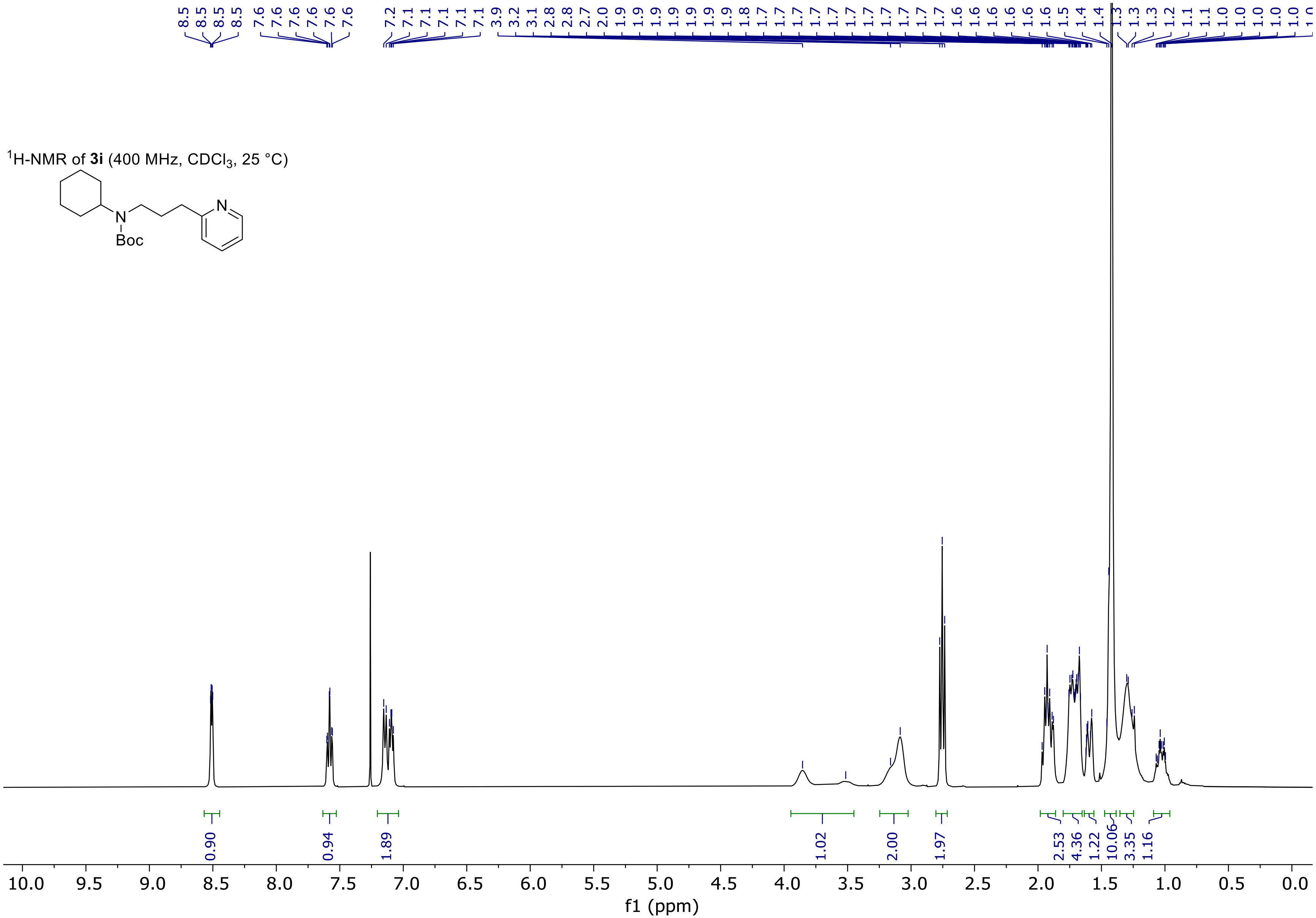
1H-NMR of 3i (400 MHz, CDCl3, 25 °C)
N
Boc
N
f1 (ppm)

^{13}C-NMR of **3i** (100 MHz, $CDCl_3$, 25 °C)

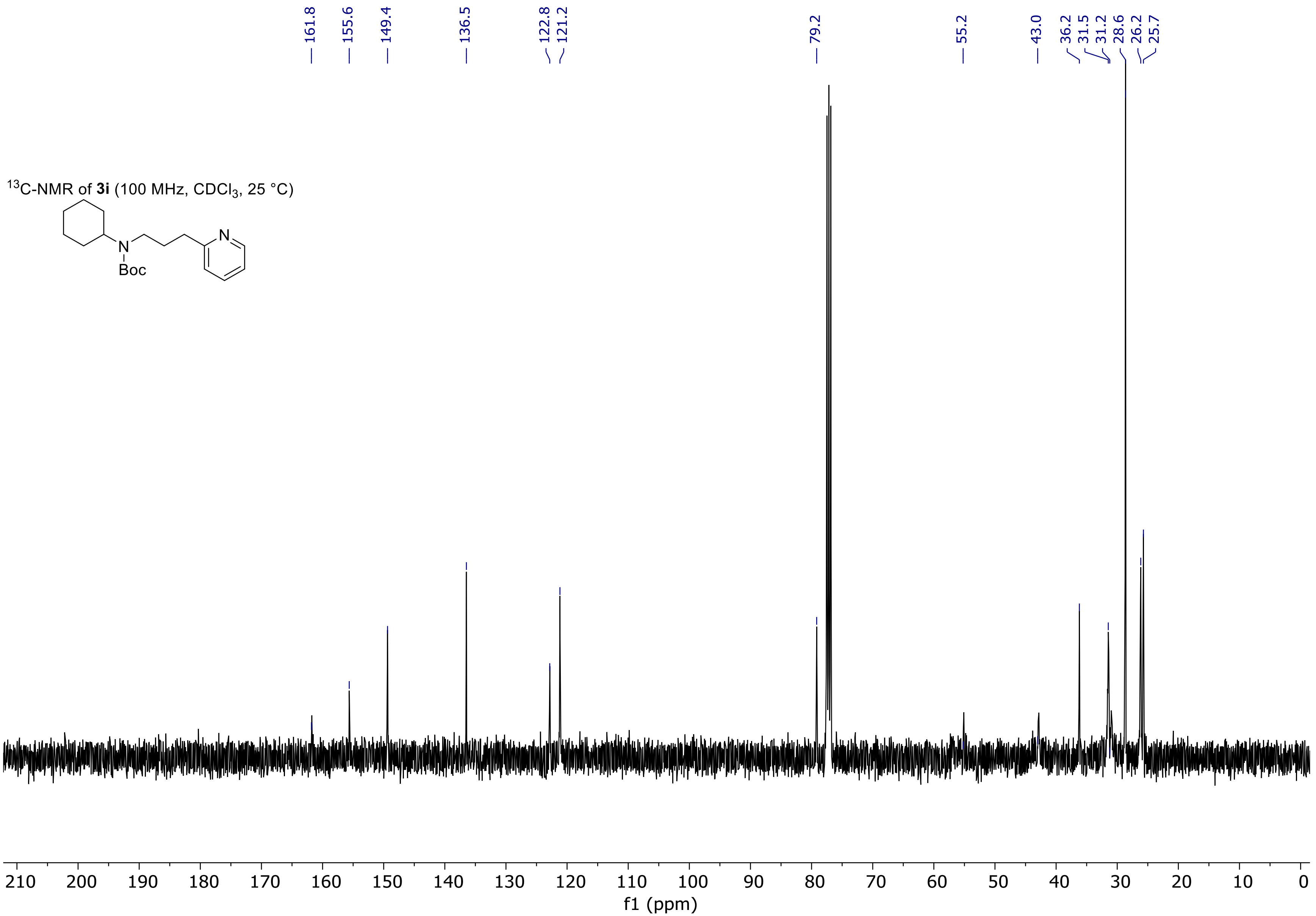

^{1}H-NMR of (±)-**3j** (400 MHz, $CDCl_3$, 25 °C)

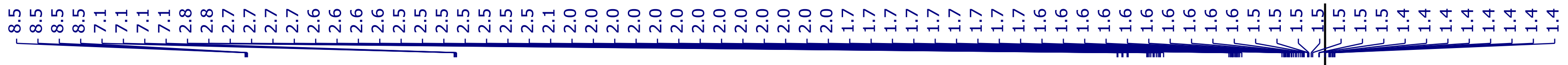

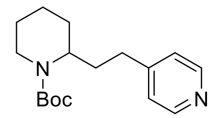

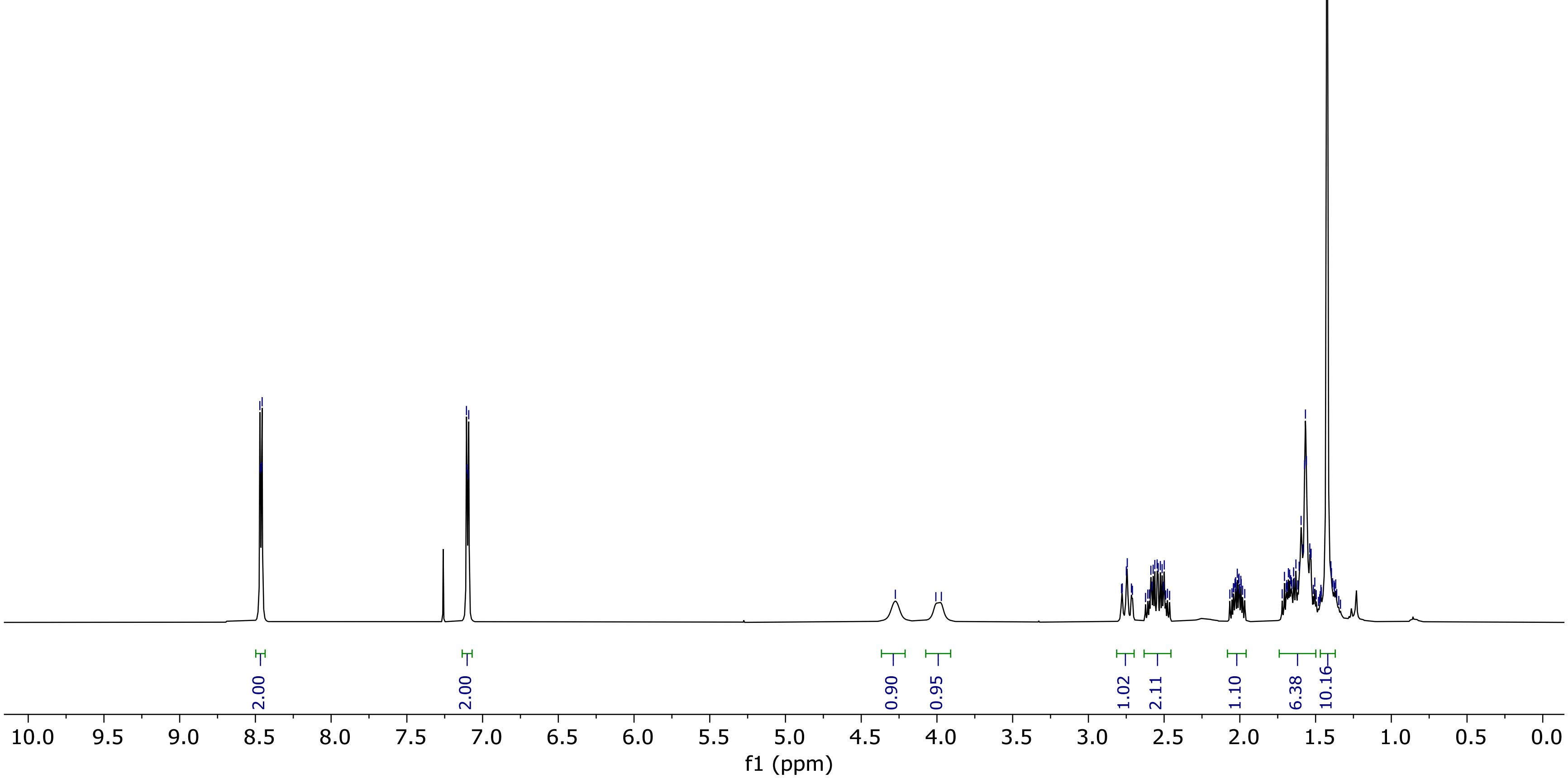

^{13}C-NMR of (±)-**3j** (100 MHz, $CDCl_3$, 25 °C)

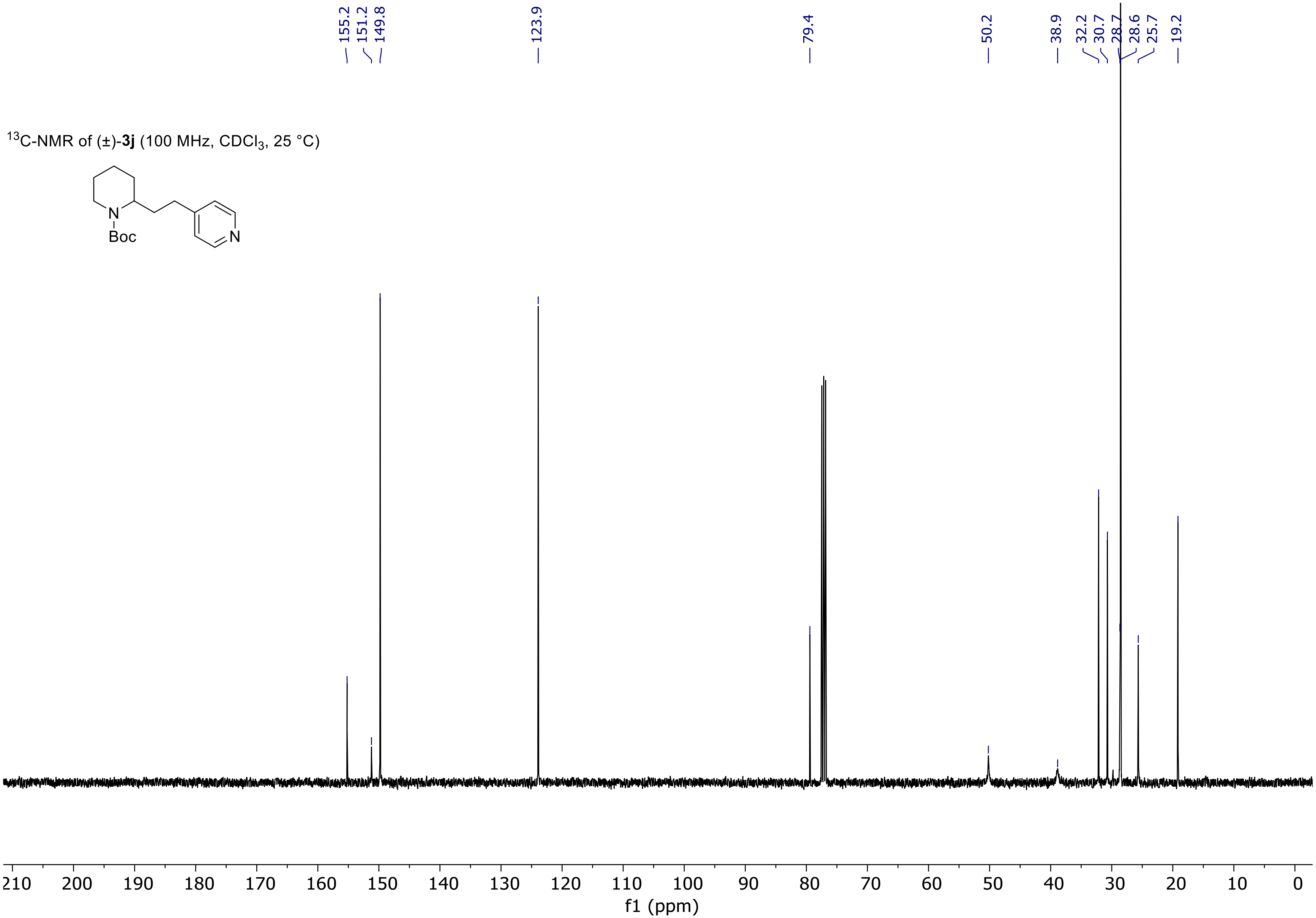

^{1}H-NMR of (±)-**3k** (400 MHz, $CDCl_3$, 25 °C)

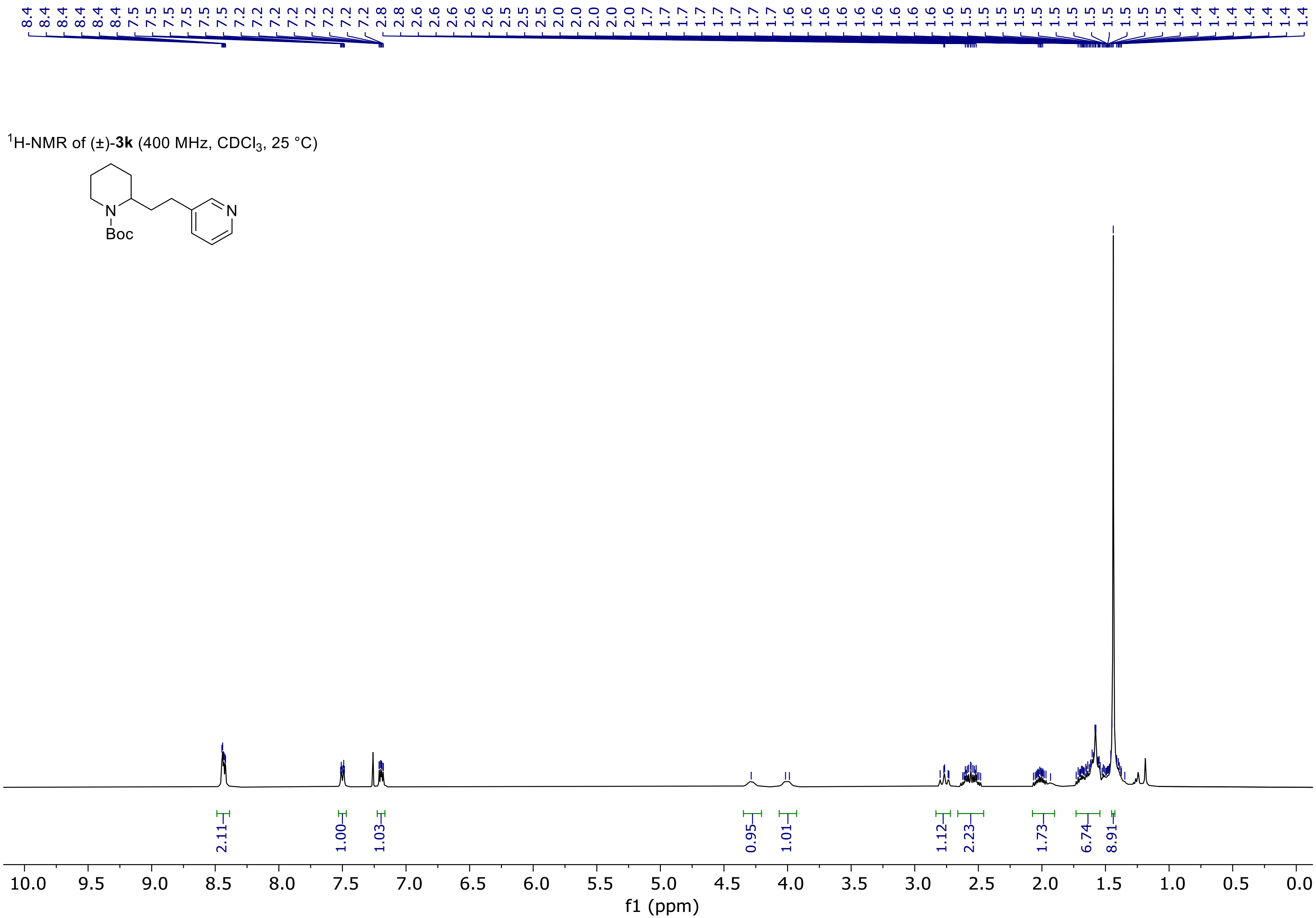

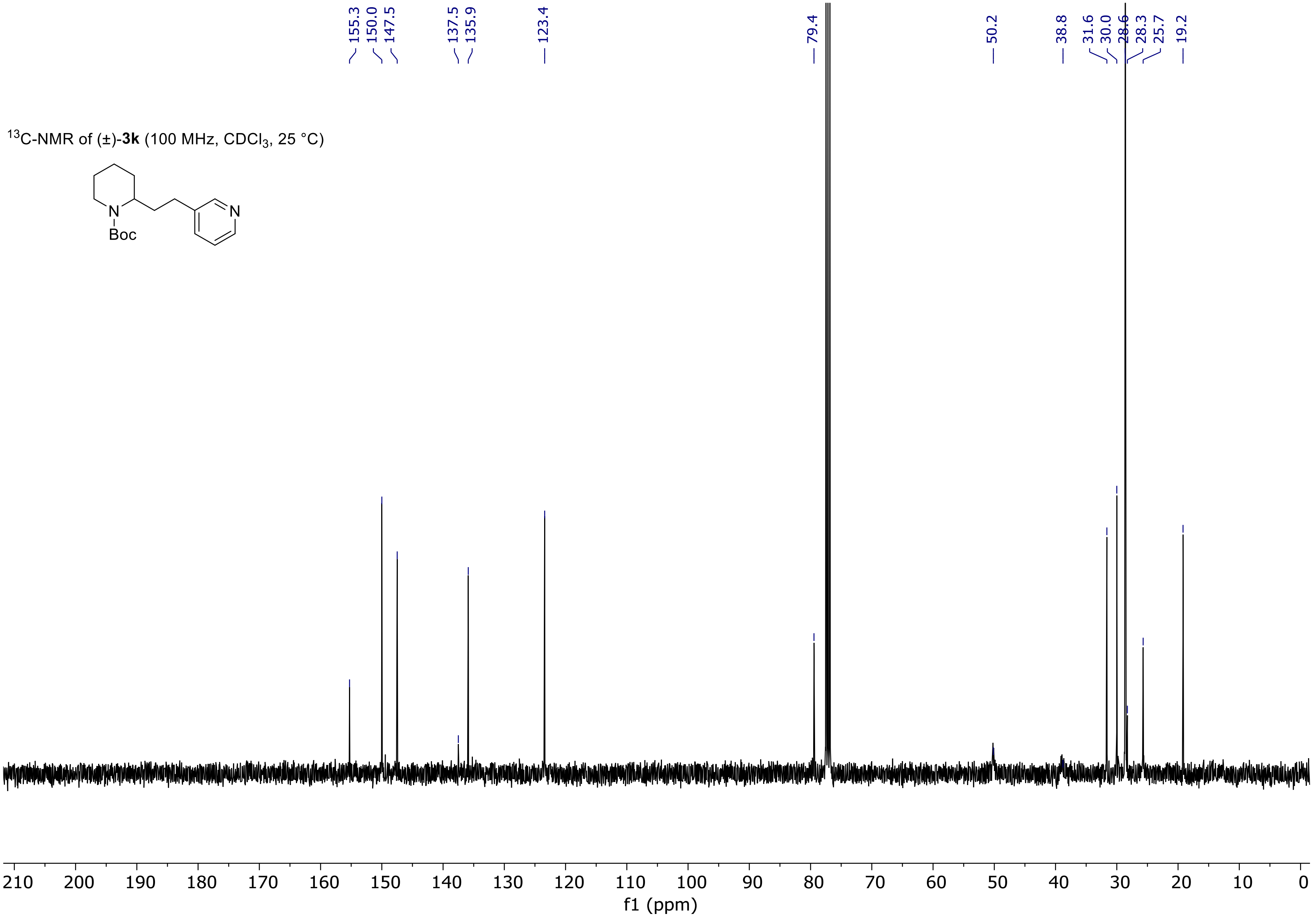

155.3
150.0
147.5
137.5
135.9
123.4
79.4
50.2
38.8
31.6
30.0
28.6
28.3
25.7
19.2
^{13}C-NMR of (±)-**3k** (100 MHz, $CDCl_3$, 25 °C)
N
Boc
N
210
200
190
180
170
160
150
140
130
120
110
100
90
80
70
60
50
40
30
20
10
0
f1 (ppm)

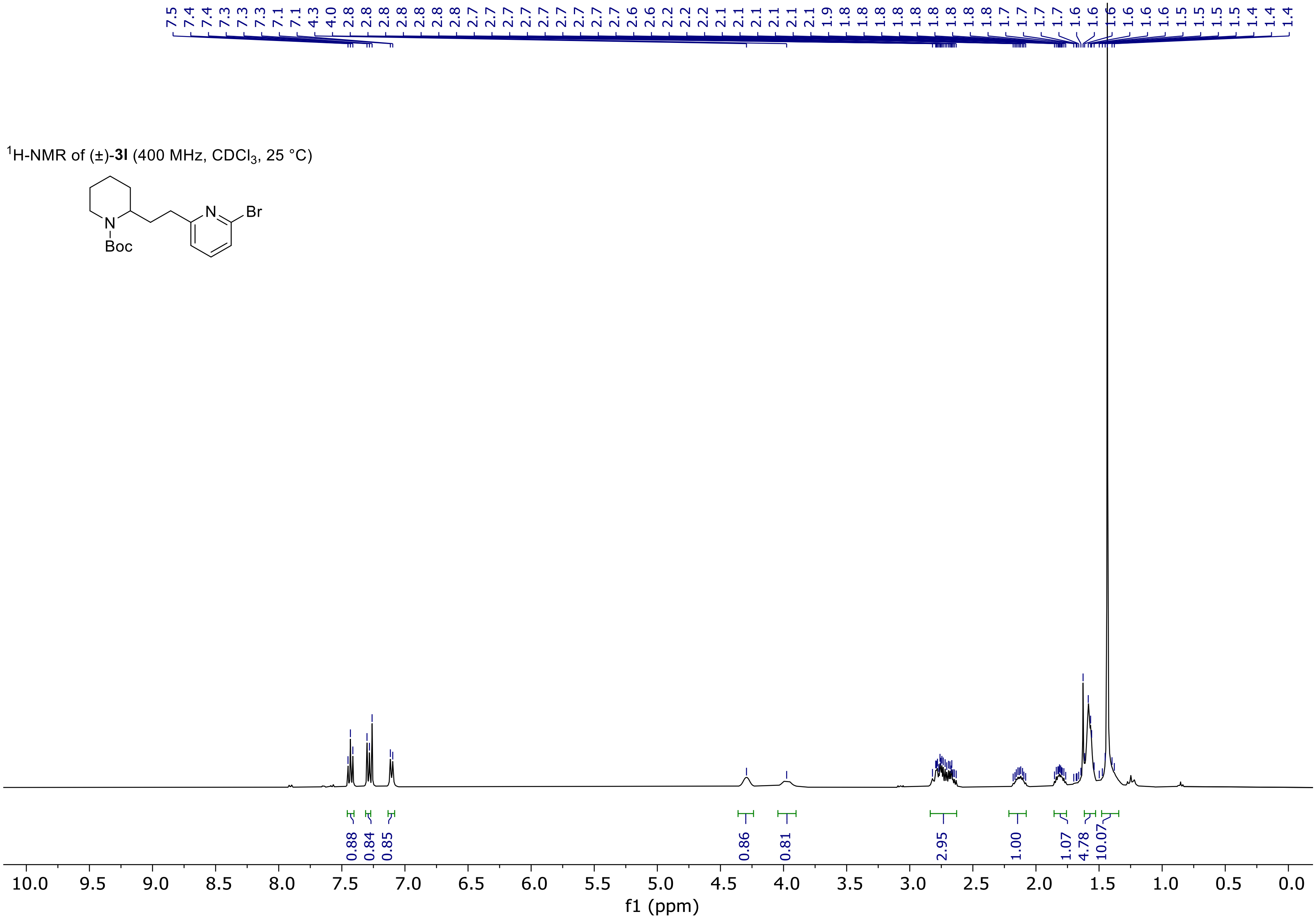

¹H-NMR of (±)-**3l** (400 MHz, CDCl₃, 25 °C)
N
Br
Boc
0.88
0.84
0.85
0.86
0.81
2.95
1.00
1.07
4.78
10.07
10.0 9.5 9.0 8.5 8.0 7.5 7.0 6.5 6.0 5.5 5.0 4.5 4.0 3.5 3.0 2.5 2.0 1.5 1.0 0.5 0.0
f1 (ppm)

^{13}C-NMR of (±)-**3l** (100 MHz, $CDCl_3$, 25 °C)

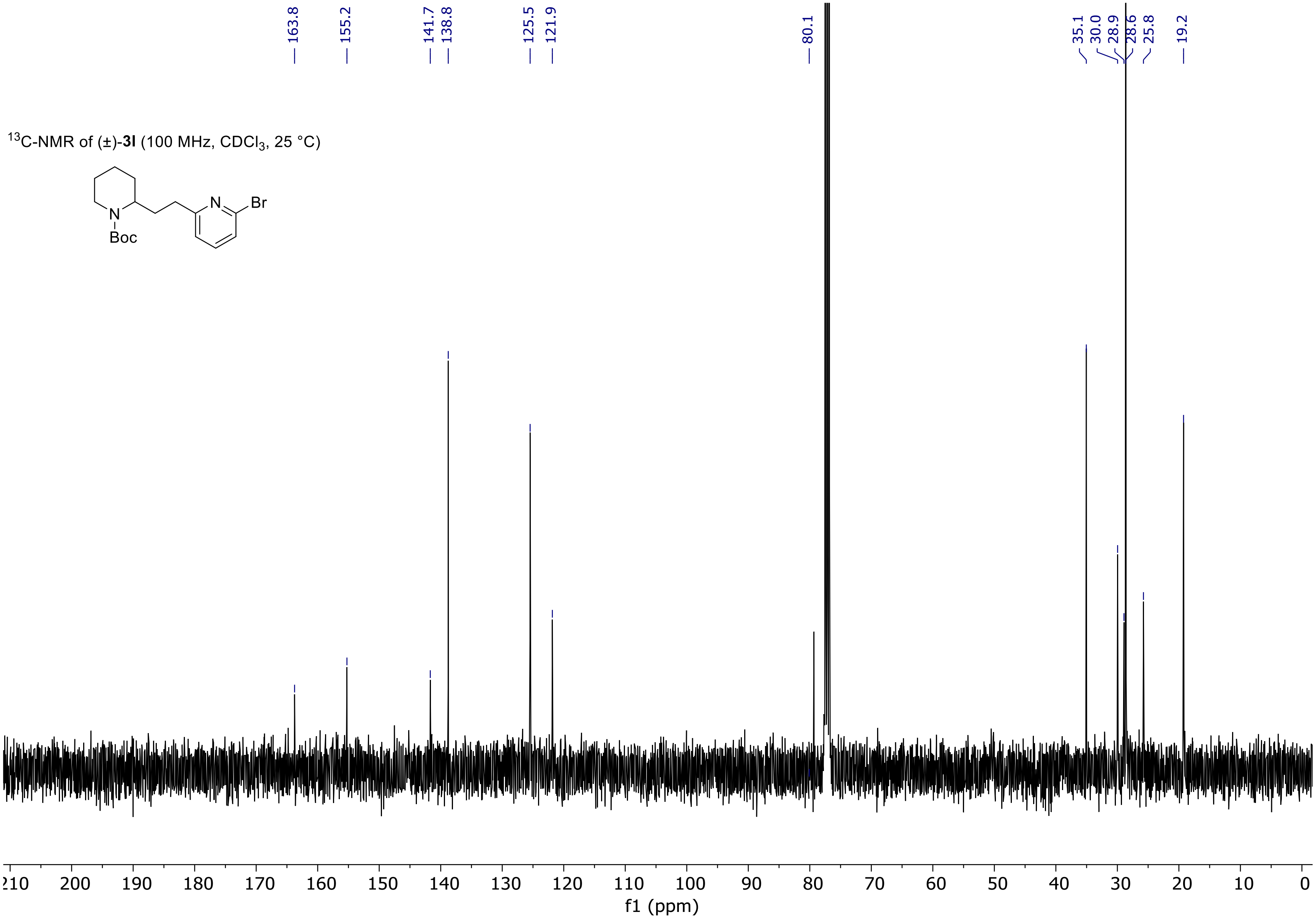

^{1}H-NMR of (±)-**3m** (400 MHz, $CDCl_3$, 25 °C)

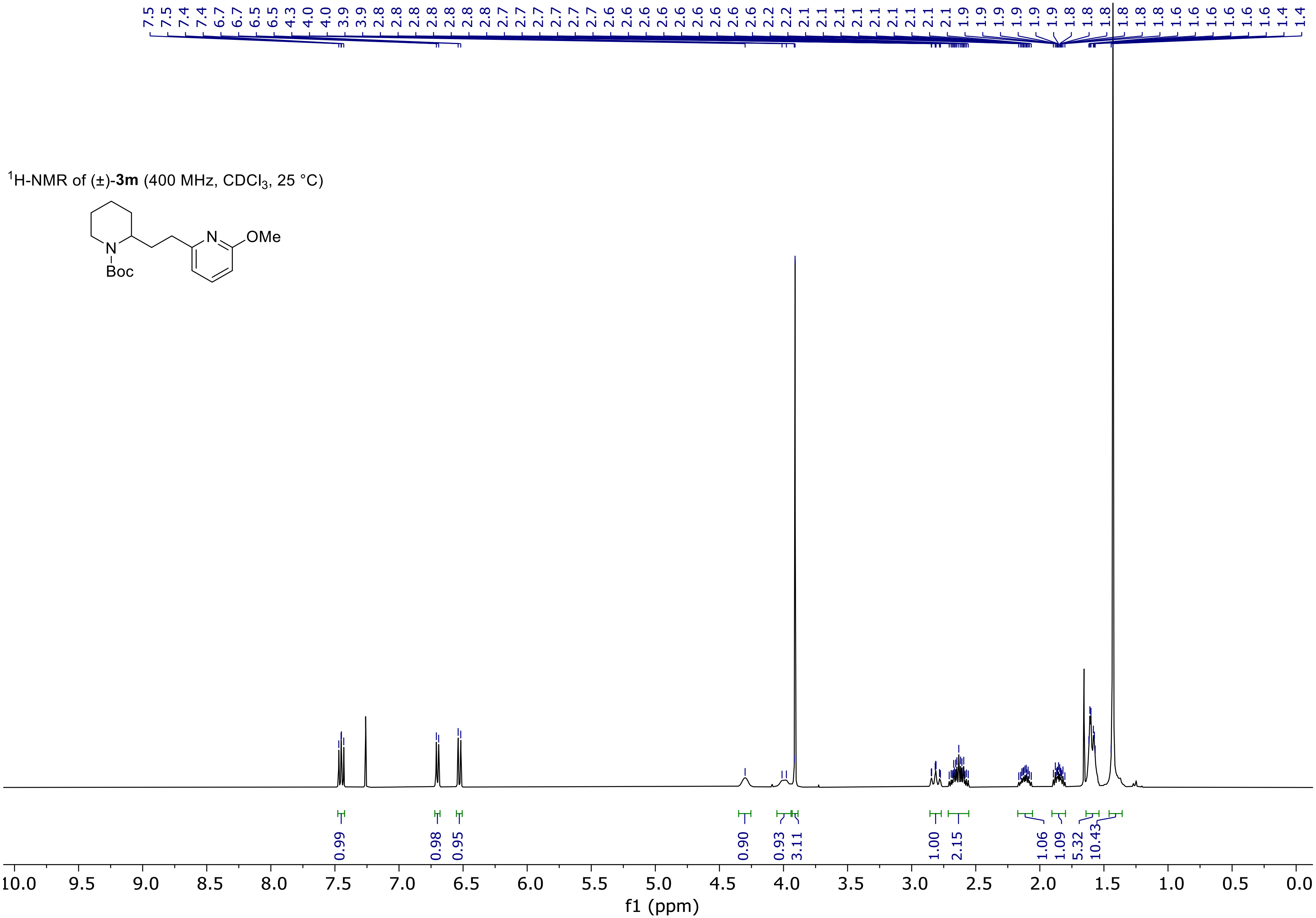

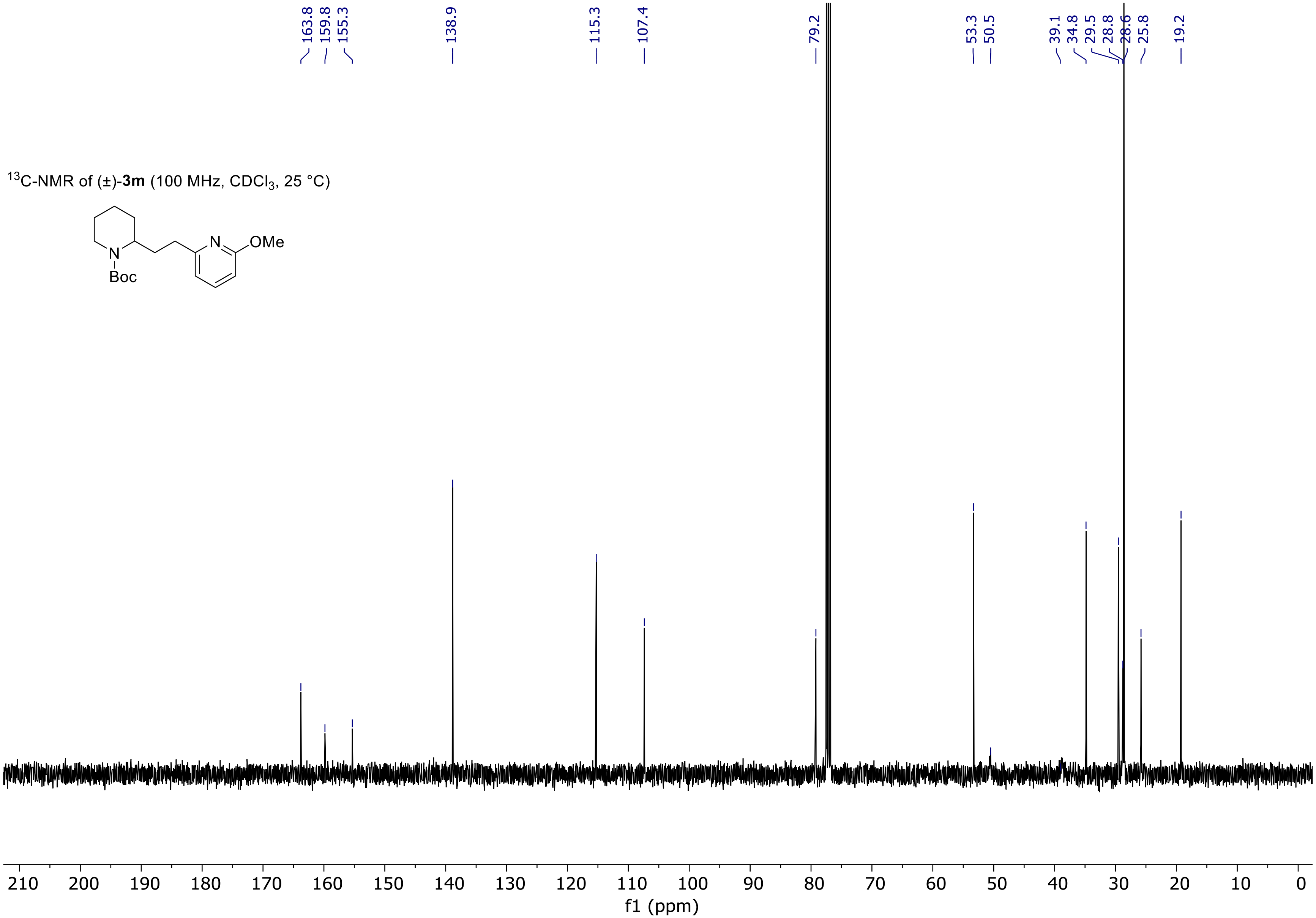

^{13}C-NMR of (±)-**3m** (100 MHz, $CDCl_3$, 25 °C)

^{1}H-NMR of (±)-**3n** (400 MHz, $CDCl_3$, 25 °C)

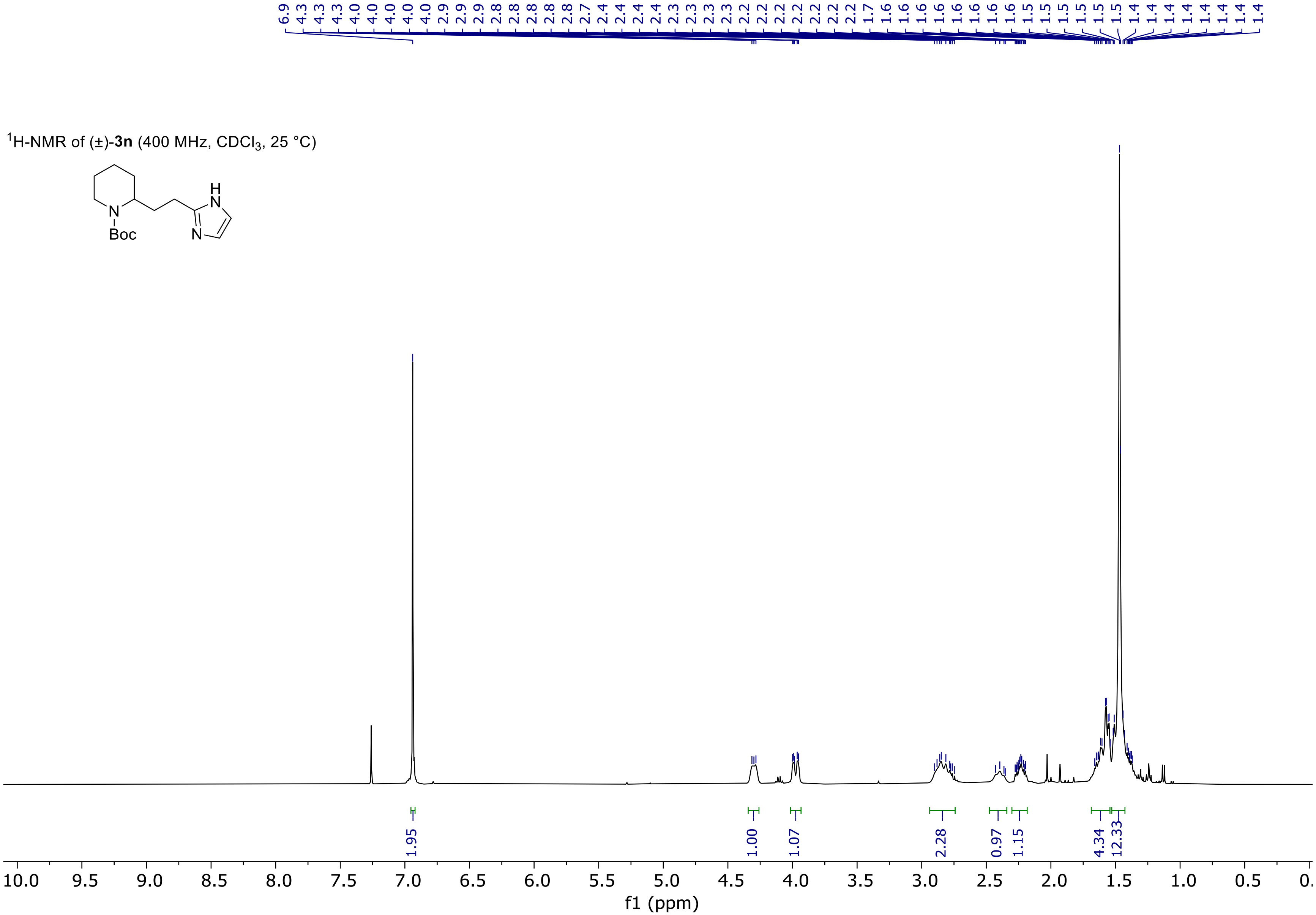

^{13}C-NMR of (±)-**3n** (100 MHz, $CDCl_3$, 25 °C)

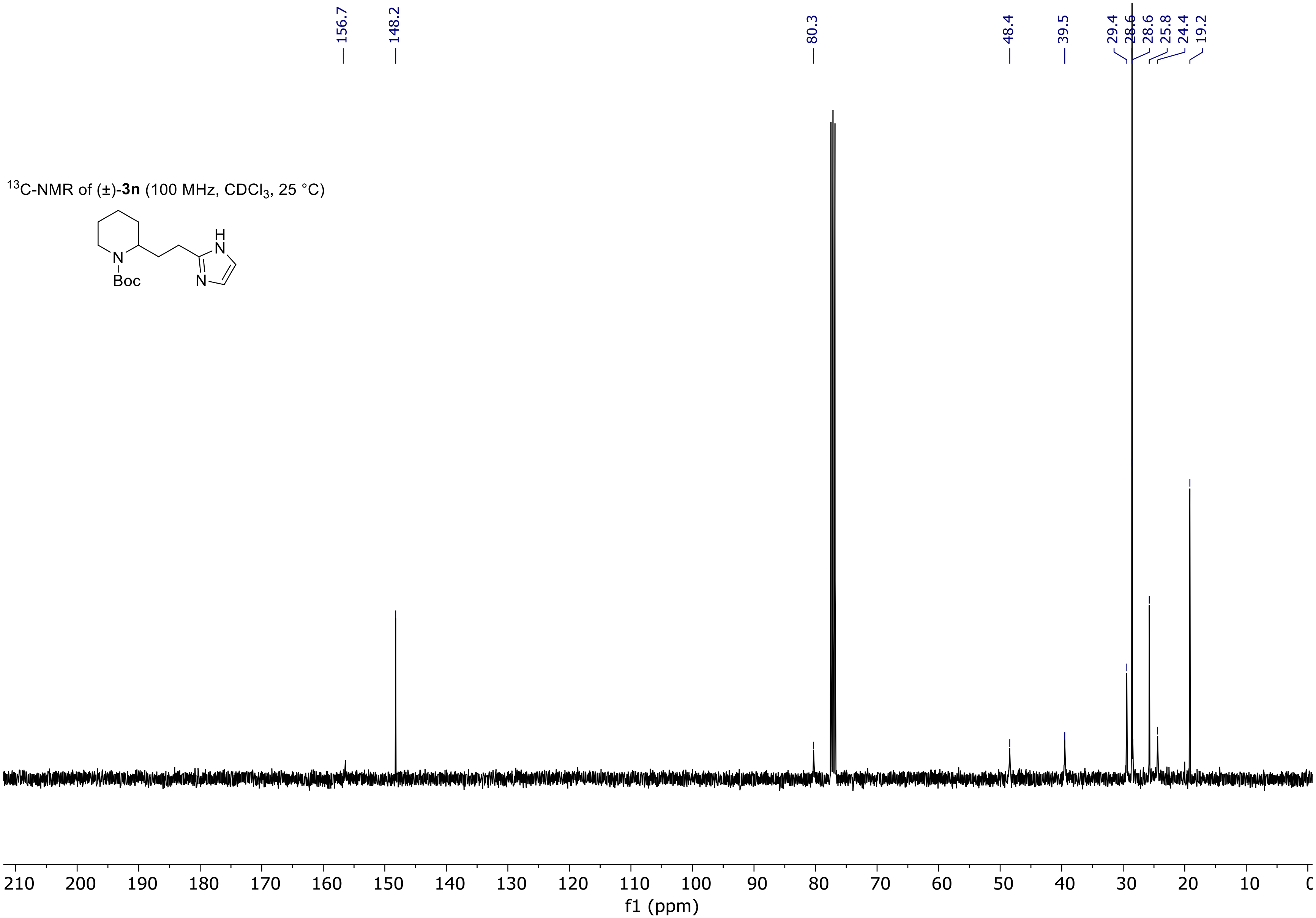

^{1}H-NMR of (±)-**3o** (400 MHz, $CDCl_3$, 25 °C)

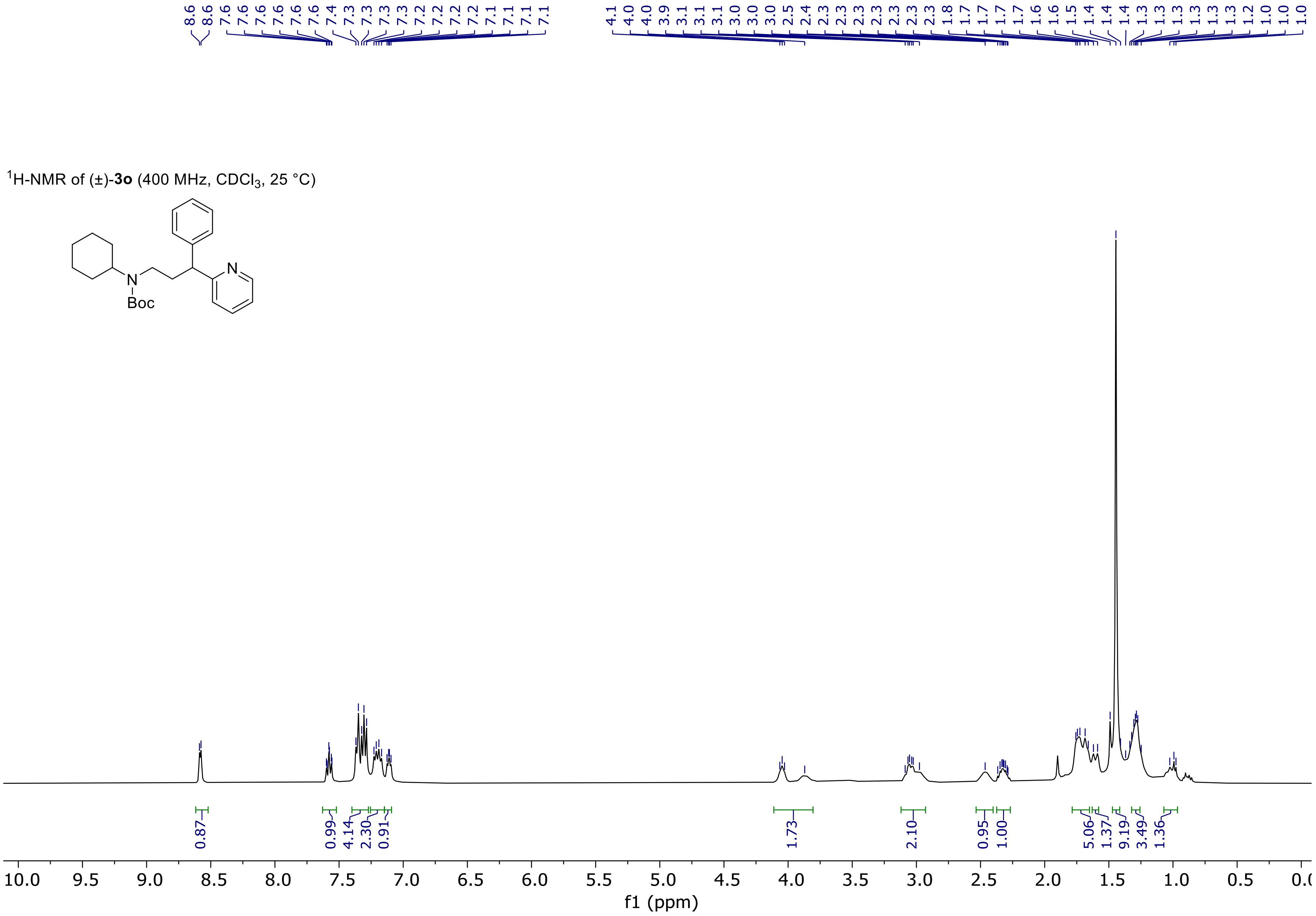

^{13}C-NMR of (±)-**3o** (100 MHz, $CDCl_3$, 25 °C)

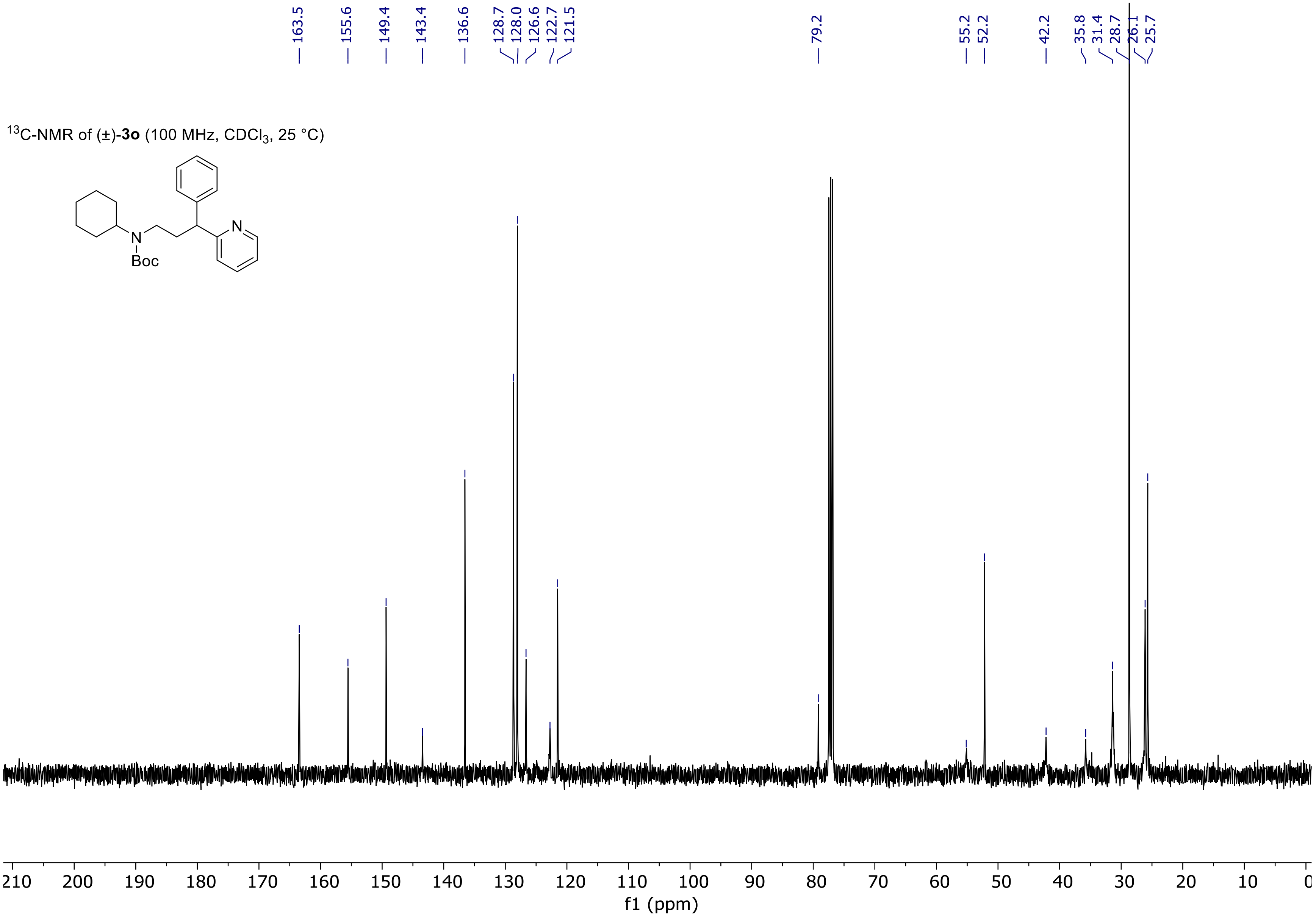

^{1}H-NMR of (±)-**5a** (600 MHz, $CDCl_3$, 25 °C)

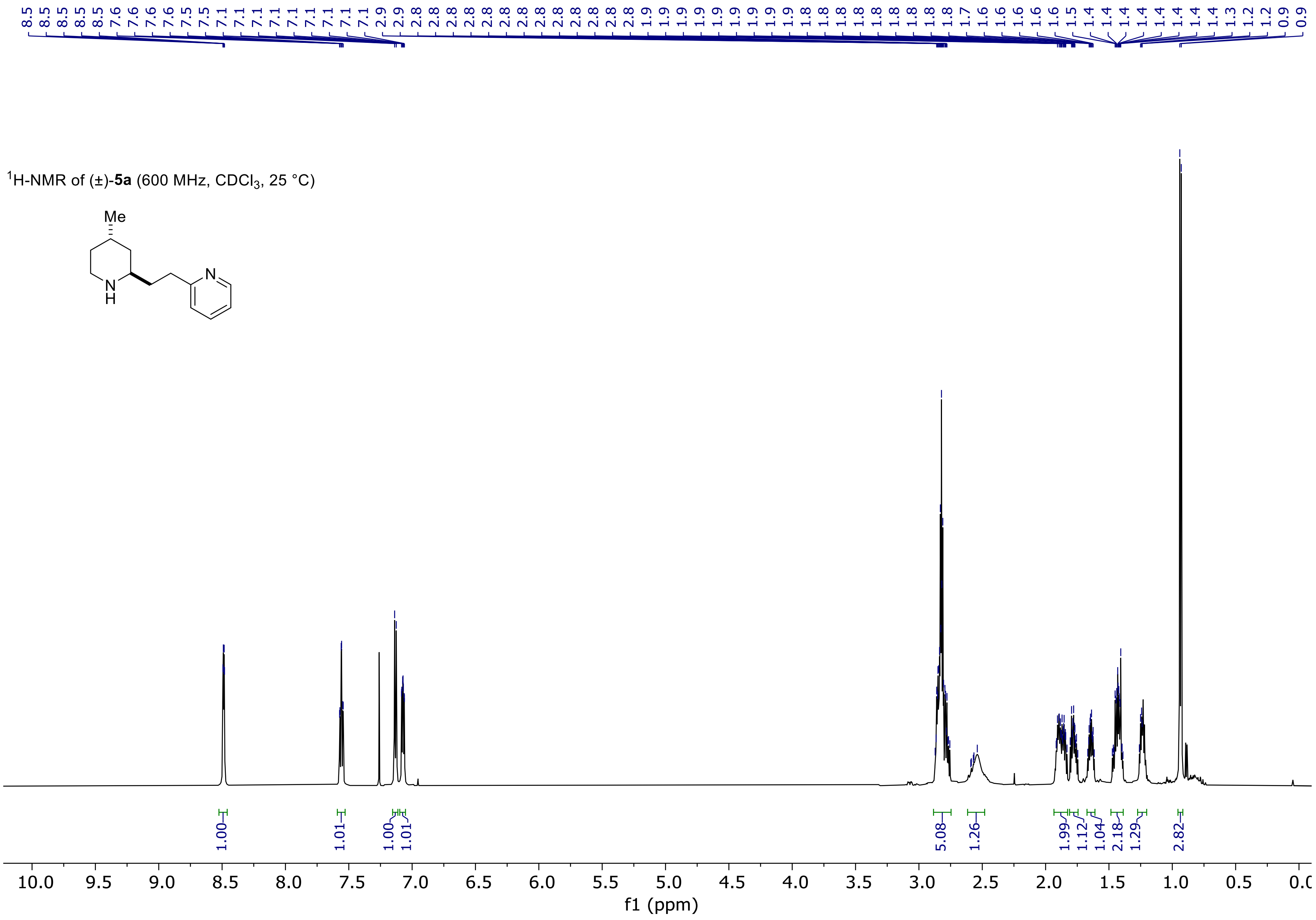

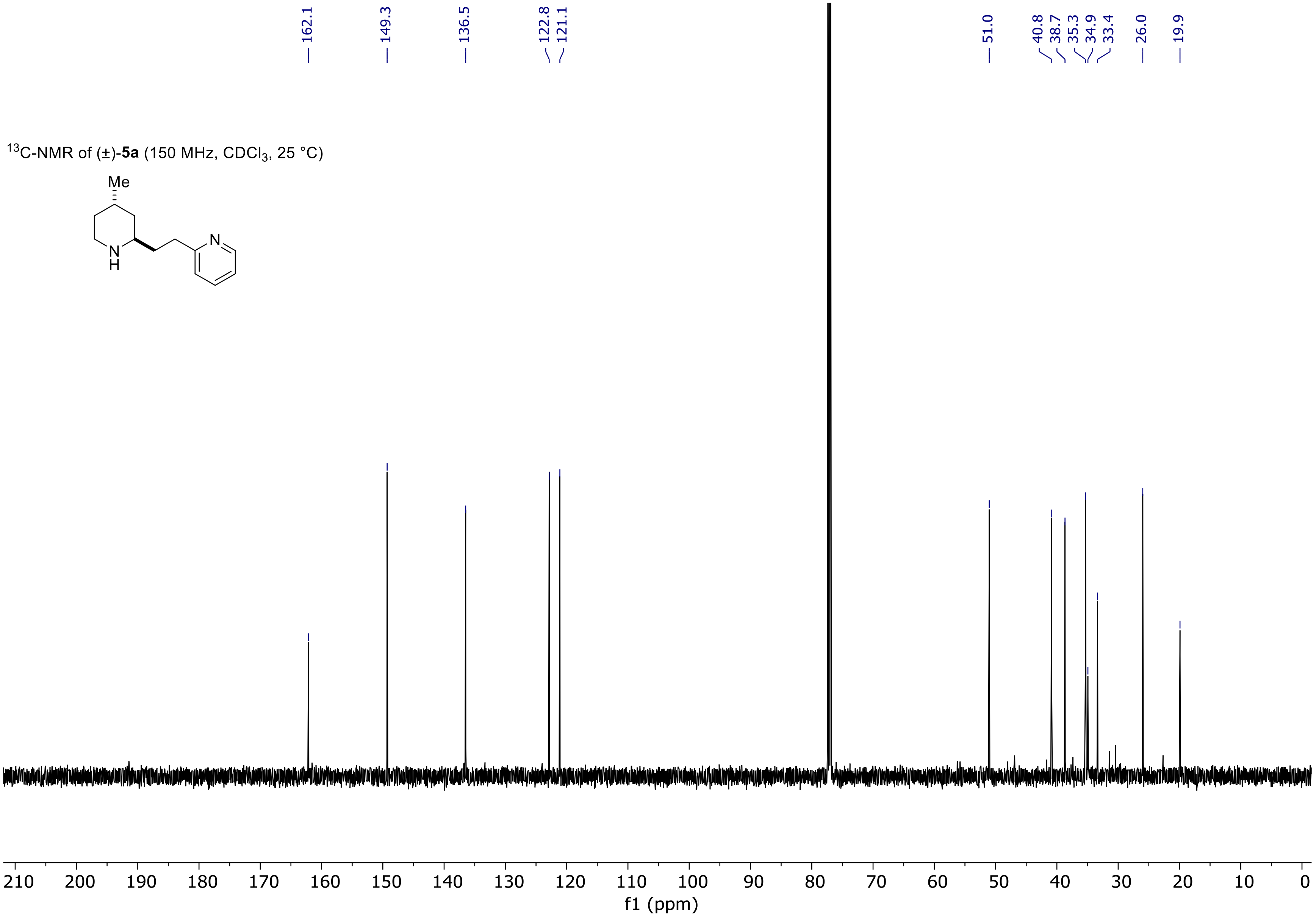
13C-NMR of (±)-5a (150 MHz, CDCl3, 25 °C)
Me
N
H
N
162.1
149.3
136.5
122.8
121.1
51.0
40.8
38.7
35.3
34.9
33.4
26.0
19.9
210 200 190 180 170 160 150 140 130 120 110 100 90 80 70 60 50 40 30 20 10 0
f1 (ppm)

^{1}H-NMR of (±)-**5b** (600 MHz, $CDCl_3$, 25 °C)

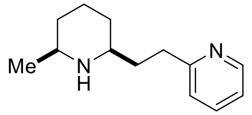

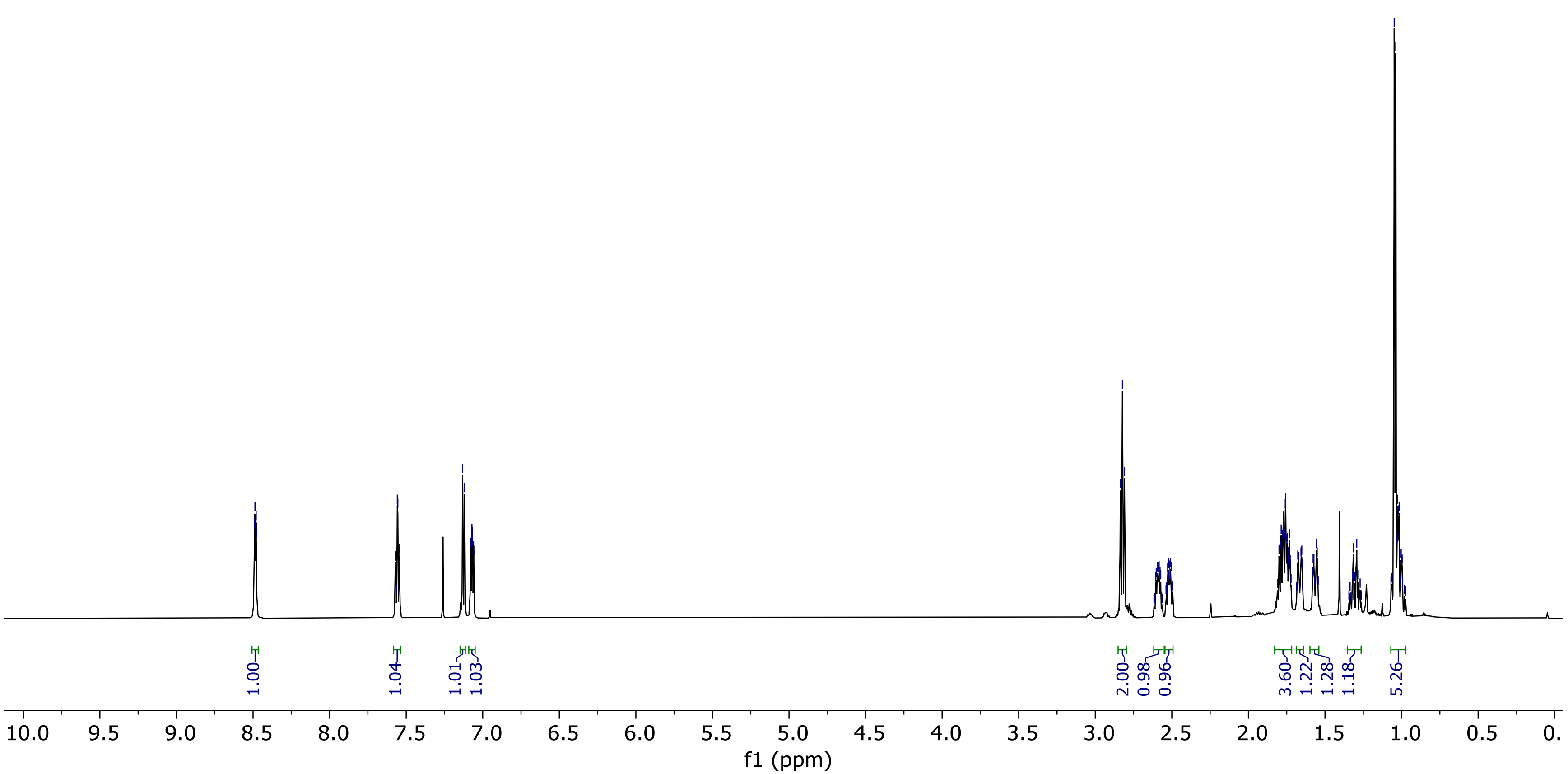

^{13}C-NMR of (±)-**5b** (150 MHz, $CDCl_3$, 25 °C)

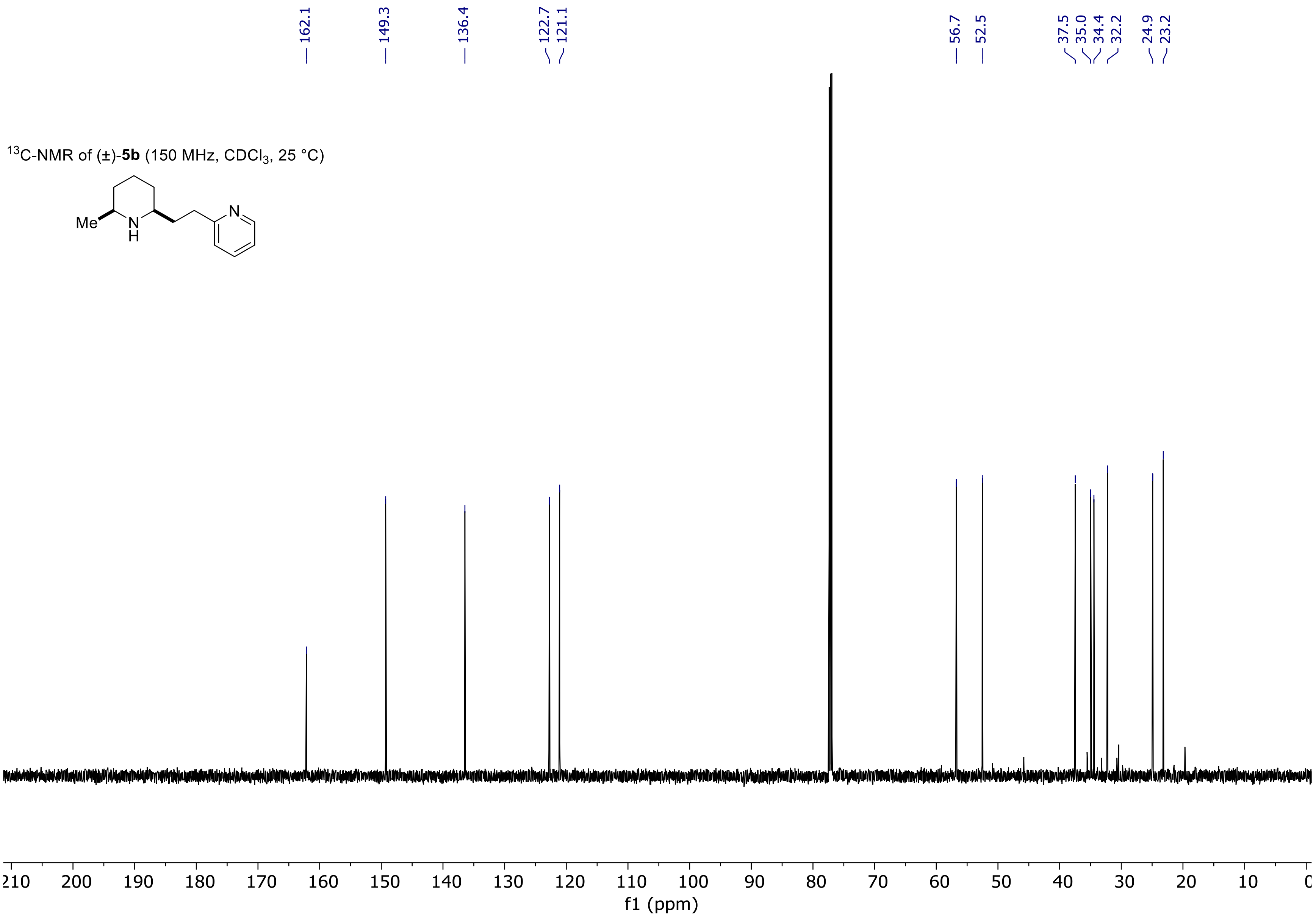

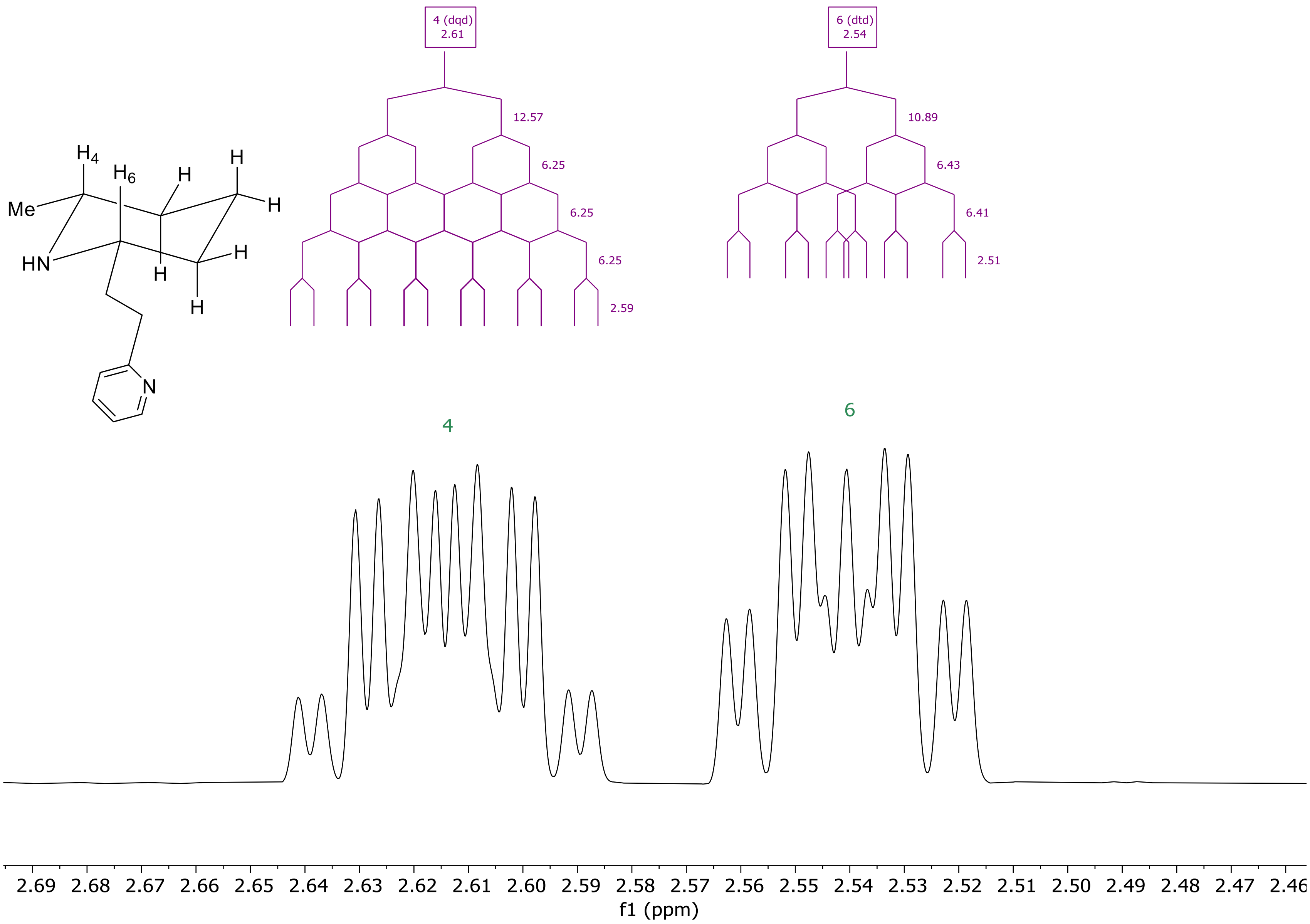

Me
HN
N
4 (dqd)
2.61
12.57
6.25
6.25
6.25
2.59
6 (dtd)
2.54
10.89
6.43
6.41
2.51
4
6
2.69 2.68 2.67 2.66 2.65 2.64 2.63 2.62 2.61 2.60 2.59 2.58 2.57 2.56 2.55 2.54 2.53 2.52 2.51 2.50 2.49 2.48 2.47 2.46
f1 (ppm)
